# Secreted Proteases Control the Timing of Aggregative Community Formation in Vibrio cholerae

**DOI:** 10.1128/mBio.01518-21

**Published:** 2021-11-23

**Authors:** Matthew Jemielita, Ameya A. Mashruwala, Julie S. Valastyan, Ned S. Wingreen, Bonnie L. Bassler

**Affiliations:** a Department of Molecular Biology, Princeton University, Princeton, New Jersey, USA; b Howard Hughes Medical Institute, Chevy Chase, Maryland, USA; c Lewis-Sigler Institute for Integrative Genomics, Princeton University, Princeton, New Jersey, USA; Max Planck Institute for Terrestrial Microbiology; University of Würzburg

**Keywords:** *Vibrio cholerae*, aggregation, biofilms, proteases, quorum sensing

## Abstract

Bacteria orchestrate collective behaviors using the cell-cell communication process called quorum sensing (QS). QS relies on the synthesis, release, and group-wide detection of small molecules called autoinducers. In Vibrio cholerae, a multicellular community aggregation program occurs in liquid, during the stationary phase, and in the high-cell-density QS state. Here, we demonstrate that this aggregation program consists of two subprograms. In one subprogram, which we call void formation, structures form that contain few cells but provide a scaffold within which cells can embed. The other subprogram relies on flagellar machinery and enables cells to enter voids. A genetic screen for factors contributing to void formation, coupled with companion molecular analyses, showed that four extracellular proteases, Vca0812, Vca0813, HapA, and PrtV, control the onset timing of both void formation and aggregation; moreover, proteolytic activity is required. These proteases, or their downstream products, can be shared between void-producing and non-void-forming cells and can elicit aggregation in a normally nonaggregating V. cholerae strain. Employing multiple proteases to control void formation and aggregation timing could provide a redundant and irreversible path to commitment to this community lifestyle.

## INTRODUCTION

Bacteria often form multicellular communities. In Vibrio cholerae, the pathogen responsible for the disease cholera, multicellular community formation is controlled by the bacterial cell-cell communication process called quorum sensing (QS). QS relies on extracellular signal molecules called autoinducers. Autoinducers are detected by the population, facilitating collective behaviors.

**FIG 1:**
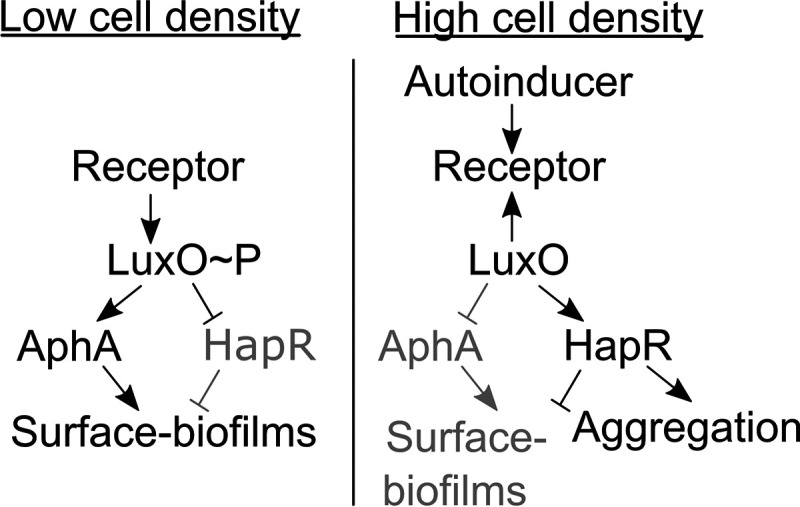
Simplified V. cholerae QS circuit. See text for details.

A simplified schematic of the V. cholerae QS circuit showing components germane to the present work is provided in Fig. 1 (1). In brief, when autoinducer concentration is low, the autoinducer receptors act as kinases that shuttle phosphate to the master response regulator LuxO. Phosphorylated LuxO, LuxO∼P, drives AphA production and represses HapR production. AphA and HapR are, respectively, the master low-cell-density (LCD) and high-cell-density (HCD) QS transcriptional regulators. When autoinducer concentration is high, the receptors act as phosphatases that promote the removal of phosphate from LuxO. Dephosphorylated LuxO is inactive, so AphA is no longer made and HapR production is no longer repressed. HapR activates expression of genes in the HCD QS regulon.

In the LCD QS state, V. cholerae forms surface biofilms, bacterial communities bound to each other and to surfaces by an extracellular matrix. In the HCD QS state, V. cholerae disperses from surface biofilm communities and reenters the planktonic, individual-cell lifestyle (2, 3). To carry out the present work, we used V. cholerae strains locked in the LCD and HCD QS modes. The strain locked in the LCD QS mode carries the *luxO D61E* mutation, encoding a LuxO phosphomimetic. The strain locked in the HCD QS mode carries the *luxO D61A* mutation, encoding a LuxO variant incapable of being phosphorylated (4).

We previously reported a liquid-based aggregative community formation program in V. cholerae (5). This program is launched in the HCD QS state and is positively regulated by HapR (Fig. 1) (5). As noted above, and in contrast, V. cholerae surface biofilm formation occurs at LCD. Formation of surface biofilms requires Vibrio polysaccharide (VPS) production (6–8). The aggregation program does not require VPS. Maturation of surface biofilms depends on cell division and takes many hours. From inception to completion, the V. cholerae aggregation program takes at most 30 min, precluding a cell division-driven mechanism (5). These differences suggest that distinct mechanisms underlie surface biofilm formation and aggregation. Previously, we performed a genetic screen to identify factors promoting aggregation. That screen revealed that motility and stress response genes, among others, are required (5).

Here, we further investigate the V. cholerae aggregation program. To ensure that we study the process independently of surface biofilm formation, unless otherwise noted, all strains lack *vpsL*, a gene essential for VPS production (9). We show that the V. cholerae aggregation program involves structures that we call voids. Voids can form in V. cholerae strains in which the flagellar machinery is disrupted. Voids contain few cells. Additionally, if all cells are removed from a culture shortly before the onset of void formation, voids still form in their absence. Thus, voids presumably provide a scaffold within which cells embed to form aggregative communities. We demonstrate that the onset timing of void formation and, therefore, the onset timing of aggregation are controlled by four extracellular proteases, Vca0812, Vca0813, HapA, and PrtV. Using site-directed mutagenesis of the Vca0812 protease as a test case, we show that proteolytic activity is necessary for proper void/aggregation onset timing. The four proteases can be shared among void-forming and non-void-forming strains. Indeed, protease-deficient V. cholerae strains that exhibit delayed void formation and aggregation timing were restored to wild-type aggregation timing by incubation with protease-harboring strains. We propose a model in which proteases cleave a substrate or substrates, converting a precursor into a product that promotes void formation/aggregation or one that loses the ability to repress void formation/aggregation. Possibly, the involvement of four redundant proteases ensures the rapid and reliable execution of this V. cholerae multicellular program.

## RESULTS

### The V. cholerae aggregation program relies on a void formation subprogram.

**FIG 2:**
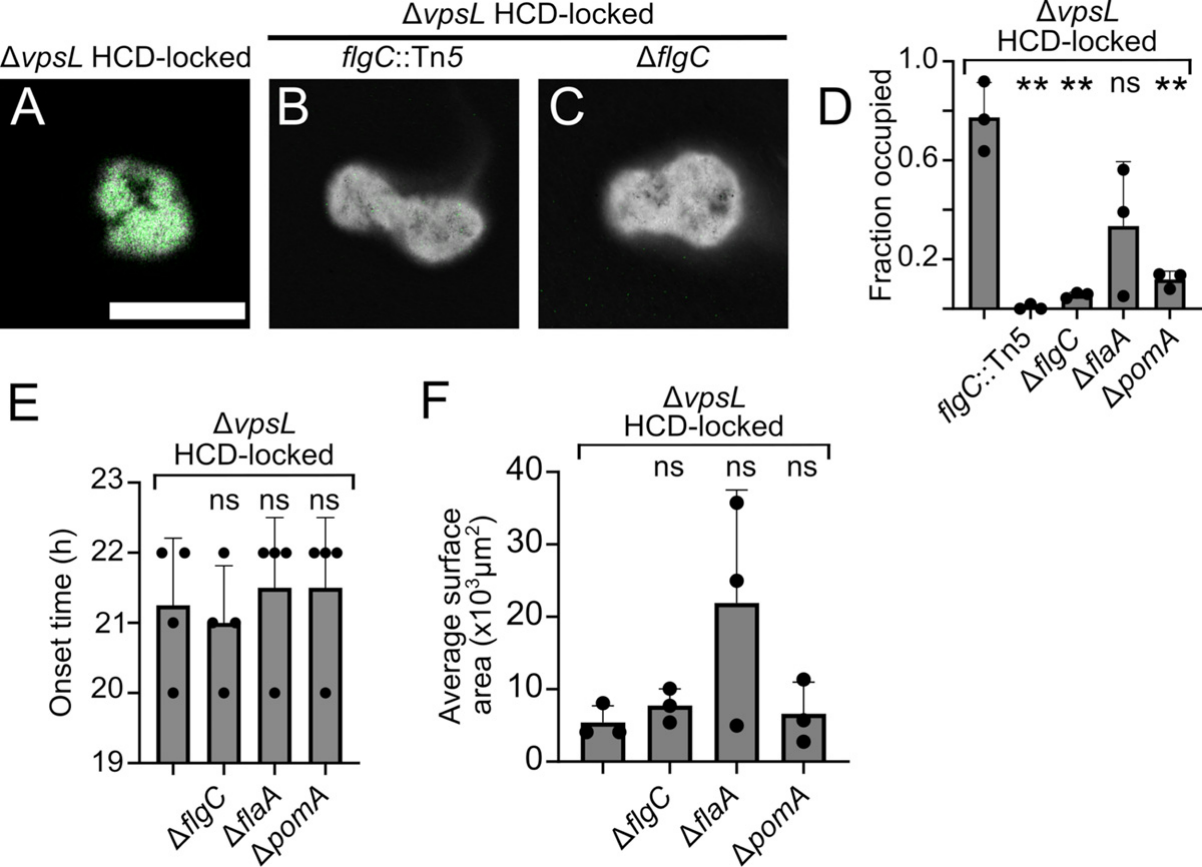
The V. cholerae aggregation program contains a void formation subprogram. Representative cross-sectional images of the designated aggregating (A) and void-forming (B, C) strains. (A to C) Treated with India ink counterstain (gray; inverted lookup table). Green, SYTO-9 nucleic acid stain. Bar, 100 μm. Magnification, ×63. (D) Quantitation of average occupancy of voids or aggregates at time (*T*) = 22 h. Error bars denote mean ± standard deviation (SD), *N* = 3 biological replicates. (E) Quantitation of onset time of designated strains. Error bars denote mean ± SD, *N* = 4 biological replicates. (F) Quantitation of average cross-sectional surface area for the strains shown in panel E. Error bars denote mean ± SD, *N* = 3 biological replicates. (B to F) All strains except the *flgC*::Tn*5* Δ*vpsL* HCD-locked mutant constitutively expressed *mKO* from the chromosome. (D to F) Statistics computed using an unpaired two-tailed *t* test comparing each data set to the Δ*vpsL* HCD-locked mutant. **, *P* < 0.005; ns, not significant.

We previously performed a transposon mutagenesis screen in the Δ*vpsL* HCD-locked V. cholerae strain to identify components required for aggregation (5). This screen revealed that a *flgC*::Tn*5* Δ*vpsL* HCD-locked mutant did not participate in aggregative community formation, in contrast to its Δ*vpsL* HCD-locked parent strain (Fig. 2A), but rather formed structures in liquid with few embedded cells (Fig. 2B). *flgC* encodes a flagellar basal body rod protein (10). We call the structures made by this mutant “voids.” Voids can be visualized using India ink negative staining (11) (Fig. 2A to C). We validated the phenotype of the *flgC*::Tn*5* Δ*vpsL* HCD-locked mutant by constructing an in-frame deletion of *flgC* to generate the Δ*flgC* Δ*vpsL* HCD-locked strain (Fig. 2C). Indeed, relative to the Δ*vpsL* HCD-locked parent strain, both the strain with the transposon insertion and the strain with the deletion in *flgC* formed voids that largely lacked embedded cells (Fig. 2D). This finding suggested that void formation is an aspect of the overall aggregation program.

To study the features of void formation and aggregation, we focused on two readily assayable phenotypes, onset timing and structure size. Every 1 h, we imaged the Δ*flgC* Δ*vpsL* HCD-locked and Δ*vpsL* HCD-locked strains, each of which contained a chromosomally integrated fluorescent *mKO* reporter. Void formation and aggregation, respectively, occurred with similar timing in the two strains (Fig. 2E), and the voids and aggregates were comparable in size (Fig. 2F).

We tested whether the inability of cells to enter voids was specific to the Δ*flgC* Δ*vpsL* HCD-locked mutant or, alternatively, whether possessing a flagellum and/or being motile was required. For this analysis, we examined two additional strains, the Δ*flaA* Δ*vpsL* HCD-locked and Δ*pomA* Δ*vpsL* HCD-locked strains. *flaA* encodes an essential flagellum subunit. Thus, Δ*flaA* mutants have no flagella. *pomA* encodes the stator complex of the flagellar motor and is required for flagellar rotation (12). Thus, Δ*pomA* mutants have flagella, but they do not rotate. Void formation timing was the same for all of these strains (Fig. 2E). The voids formed by the Δ*flaA* Δ*vpsL* HCD-locked strain harbored more embedded cells than the voids formed by the Δ*pomA* Δ*vpsL* HCD-locked and Δ*flgC* Δ*vpsL* HCD-locked strains, but harbored fewer cells than the voids formed by the Δ*vpsL* HCD-locked parent (Fig. 2D). The Δ*flaA* Δ*vpsL* HCD-locked strain also made larger structures than those made by the other strains (Fig. 2F). Complementation of the Δ*flgC* Δ*vpsL* HCD-locked, Δ*flaA* Δ*vpsL* HCD-locked, and Δ*pomA* Δ*vpsL* HCD-locked strains with the corresponding genes introduced at an ectopic locus and driven by an inducible promoter restored aggregate formation (see Fig. S1A in the supplemental material). We conclude that V. cholerae void formation does not require flagella or motility. However, flagella and motility contribute to aggregate formation, presumably by facilitating cell entrance into voids, although additional factors may also contribute. We do not yet know if motility *per se* is required, or rather, if flagellar rotation plays a regulatory or adhesive role in aggregate formation. Additionally, there appears to be FlaA-mediated regulation of void size and cell entry (Fig. 2D and F). We note that mutation of *flaA* caused pleotropic effects in other studied V. cholerae strains (13). We do not investigate the FlaA-mediated effect further in the current work.

10.1128/mBio.01518-21.1 FIG S1 Complementation restores wild-type aggregation phenotypes. (A) Quantitation of average void or aggregation occupancy, assayed between time (*T*) = 25 and 28 h. Arabinose (0.05% wt/vol) was present throughout the growth of the designated strains. All strains constitutively expressed *mKO* from the chromosome. Unpaired two-tailed *t* test compared each strain with and without arabinose supplementation. (B) Quantification of aggregation onset timing delay for the designated strains relative to that of the Aggregate^+^ strain. Strains were visualized using the fluorescent stain SYTO-9. Unpaired two-tailed *t* test comparing measured onset time delay difference to a mock experiment with no onset timing delay between the complemented and uncomplemented strains. (A, B) *, *P* < 0.05; ns, not significant. Error bars denote mean ± standard deviation (SD), *N* = 3 biological replicates. Download FIG S1, TIF file, 2.4 MB. Copyright © 2021 Jemielita et al. 2021 Jemielita et al. https://creativecommons.org/licenses/by/4.0/ This content is distributed under the terms of the Creative Commons Attribution 4.0 International license.

Based on the above findings, we propose that the aggregation program consists of the following two subprograms: one subprogram is void formation, in which structures are made within which cells can embed, and the other subprogram uses the flagellar machinery to facilitate cell entry into voids to form aggregates. Here, we used the Δ*flgC* Δ*vpsL* HCD-locked strain as our model strain that only engages in the void formation subprogram. We call the Δ*flgC* Δ*vpsL* HCD-locked strain the “Void^+^” strain. We call the Δ*vpsL* HCD-locked strain that undergoes the full aggregation program the “Aggregate^+^” strain.

### A genetic screen identifies V. cholerae factors that promote void formation.

We reasoned that we could identify factors contributing to void formation by mutagenizing the Void^+^ strain and screening for defects in this process. To accomplish this, we needed to rapidly distinguish void-forming from non-void-forming mutants. The Aggregate^+^ and the Void^+^ strains can be differentiated from the nonaggregating Δ*vpsL* LCD-locked strain based on colony morphology. On agar plates, Void^+^ and Aggregate^+^ colonies are opaque, while nonaggregating Δ*vpsL* LCD-locked colonies are translucent (Fig. S2). We do not know why colony opacity correlates with the ability to form aggregates and voids; however, the phenotype facilitated our genetic screen. We mutagenized the Void^+^ strain with Tn*5* and screened ∼65,000 colonies for those that obtained translucent phenotypes, suspecting that some could be non-void-forming mutants. We used a secondary microscopy screen to assess void formation. Our strategy yielded 92 putative candidates with mutations mapping to 25 loci (Table S1). The mutated genes encoded the regulatory proteins RpoS, CyaA, HapR, VarS, and SspA and biosynthetic and metabolic enzymes, among others. Here, we focus on one identified operon, *vca0812*-*vca0813*, that encodes two putative extracellular proteases, Vca0812 and Vca0813 (14, 15). *vca0812* and *vca081*3 are occasionally called *lap* and *lapX* (15, 16). Here, we first study Vca0812 and Vca0813 in the Void^+^ background to determine how they contribute to void formation, and second, we assess their roles in the Aggregate^+^ background to define their effects on aggregation.

10.1128/mBio.01518-21.2 FIG S2 Colony opacity phenotypes used for a genetic screen for components involved in void formation. (A) Δ*vpsL* low cell density (LCD)-locked strain (nonaggregating), (B) Aggregate^+^, and (C) Void^+^ colonies grown on Luria broth (LB) plates supplemented with 0.5% glycerol. Images taken using a smartphone camera with ambient light. Download FIG S2, TIF file, 2.2 MB. Copyright © 2021 Jemielita et al. 2021 Jemielita et al. https://creativecommons.org/licenses/by/4.0/ This content is distributed under the terms of the Creative Commons Attribution 4.0 International license.

10.1128/mBio.01518-21.9 TABLE S1 Genes identified in our genetic screen. Download Table S1, PDF file, 0.5 MB. Copyright © 2021 Jemielita et al. 2021 Jemielita et al. https://creativecommons.org/licenses/by/4.0/ This content is distributed under the terms of the Creative Commons Attribution 4.0 International license.

### The extracellular proteases Vca0812 and Vca0813 control the onset timing of V. cholerae void formation and aggregation.

**FIG 3:**
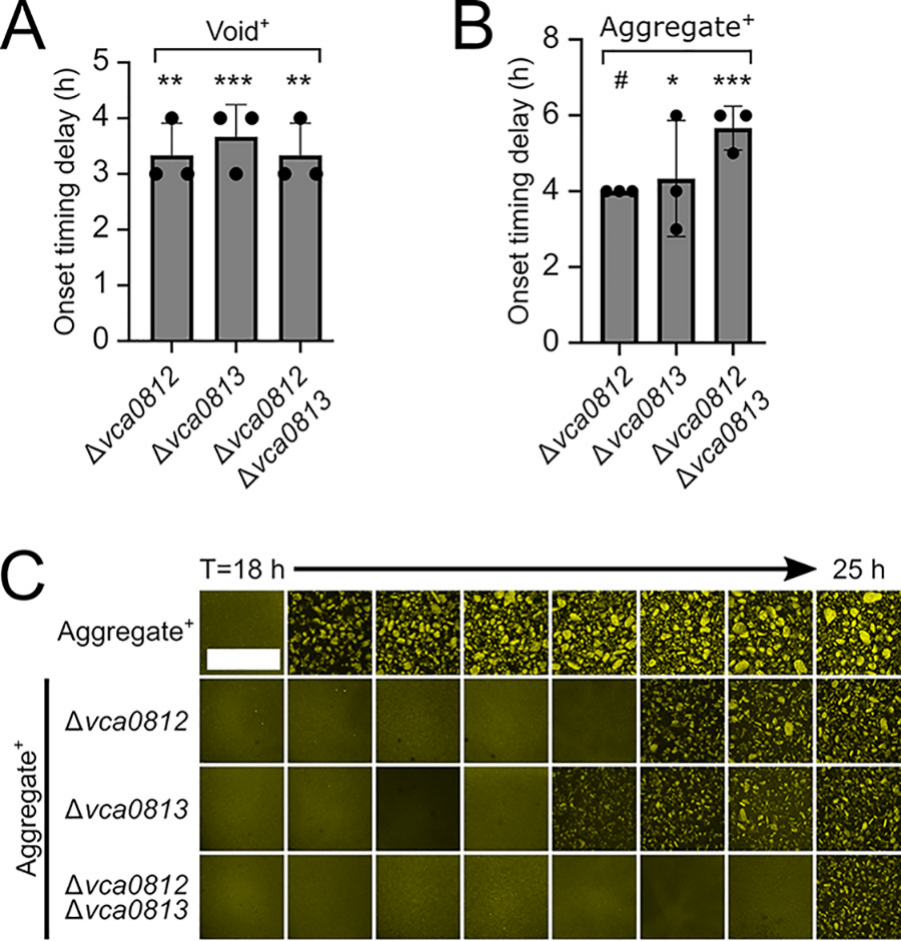
A genetic screen reveals that *vca0812* and *vca0813* encode regulators of V. cholerae void formation onset timing. (A) Quantitation of void formation onset timing delay for the designated strains relative to that of the Void^+^ strain. (B) Quantitation of aggregation onset timing delay for the designated strains relative to that of the Aggregate^+^ strain. (A, B) Error bars denote mean ± SD, *N* = 3 biological replicates. Unpaired two-tailed *t* test comparing measured onset time delay to a mock experiment with no onset timing delay. *, *P* < 0.05; **, *P* < 0.005; ***, *P* < 0.0005; ns, not significant; #, unable to estimate due to lack of data variance. (C) Representative cross sections through the designated strains imaged every 1 h. Bar, 1,000 μm. Magnification, ×10. (A to C) All strains constitutively expressed *mKO* from the chromosome.

To study the roles of Vca0812 and Vca0813 in void formation, we deleted *vca0812* and *vca0813* individually and together in the Void^+^ parent strain. We assayed the mutants for void formation capability relative to that of the parent Void^+^ strain. The strains lacking either or both proteases showed ∼3.5-h delays in void formation onset (Fig. 3A); however, ultimately, they formed voids. We conclude that Vca0812 and Vca08123 influence void formation onset timing.

To examine whether Vca0812 and Vca0813 also affect aggregation onset timing, we constructed the same deletions in the Aggregate^+^ background. Deletion of *vca0812* and/or *vca0813* resulted in aggregation onset delays of ∼4 h or more compared to the parent Aggregate^+^ strain (Fig. 3B and C). There were no growth defects in any of the strains containing the *vca0812* and *vca0813* deletions (Fig. S3A). To verify that Vca0812 and Vca0813 drive aggregation onset timing, we complemented the Δ*vca0812* Aggregate^+^ and Δ*vca0813* Aggregate^+^ strains with, respectively, *vca0812* and *vca0813* under the native promoter from an ectopic chromosomal locus. In both cases, normal aggregation timing was restored (Fig. S1B). Finally, GbpA, a V. cholerae adhesin (17, 18) that is encoded by the *vca0811* gene and located immediately adjacent to the *vca0812*-*vca0813* operon does not contribute to void formation or aggregation (Fig. S4). We conclude that Vca0812 and Vca0813 control void formation onset timing, which consequently affects aggregation onset timing. However, other components must also participate in void formation and in the aggregation program because in the absence of these two proteins, voids still form and aggregation occurs, albeit with delayed timing.

10.1128/mBio.01518-21.3 FIG S3 Growth curves for strains used in this work. (A) Growth curves for the Aggregate^+^ (red), Δ*vca0812* Aggregate^+^ (green), and Δ*vca0813* Aggregate^+^ (blue) strains. (B) Growth curves for Aggregate^+^ (red), Δ*hapA* Aggregate^+^ (green), Δ*prtV* Aggregate^+^ (blue), and Δ*4* Aggregate^+^ (black) strains. (C) Growth curves for the Aggregate^+^ strain to which no (red), a low (blue), or a high (green) concentration of a protease inhibitor cocktail was added at *T* = 0 h. (A to C) *N* = 3 biological replicates. All strains constitutively expressed *mKO* from the chromosome. Individual data traces were offset by the time at which the samples entered exponential growth. Download FIG S3, TIF file, 2.5 MB. Copyright © 2021 Jemielita et al. 2021 Jemielita et al. https://creativecommons.org/licenses/by/4.0/ This content is distributed under the terms of the Creative Commons Attribution 4.0 International license.

10.1128/mBio.01518-21.4 FIG S4 GbpA does not contribute to void formation or aggregation onset timing. Quantitation of void formation and aggregation onset timing delay for the designated strains relative to, respectively, the Void^+^ and Aggregate^+^ strains. Error bars denote mean ± SD, *N* = 3 biological replicates. All strains constitutively expressed *mKO* from the chromosome. Unpaired two-tailed *t* test comparing measured onset time delay to a mock experiment with no onset timing delay. ns, not significant; #, unable to estimate due to lack of data variance. Download FIG S4, TIF file, 2.2 MB. Copyright © 2021 Jemielita et al. 2021 Jemielita et al. https://creativecommons.org/licenses/by/4.0/ This content is distributed under the terms of the Creative Commons Attribution 4.0 International license.

### The secreted proteases HapA and PrtV also contribute to void and aggregate onset timing.

**FIG 4:**
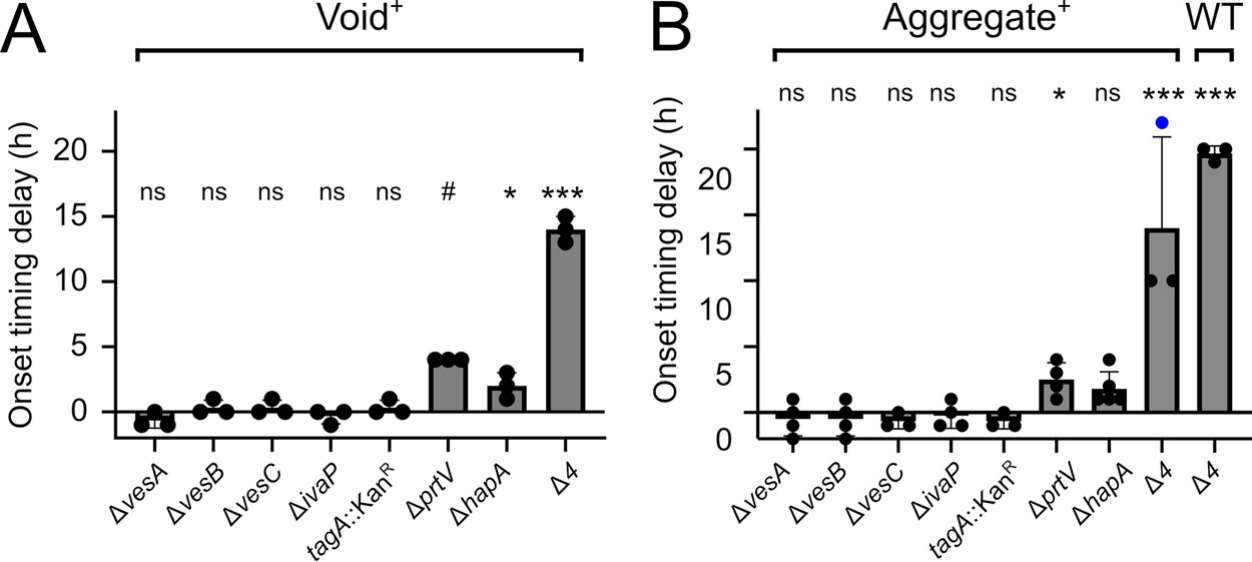
HapA and PrtV regulate V. cholerae void formation and aggregation onset timing. (A) Quantitation of void formation onset timing delay of the designated strains relative to that of the Void^+^ strain. (B) Quantitation of aggregation onset timing delay of the designated strains relative to that of the Aggregate^+^ strain. The blue circle indicates a sample that did not exhibit aggregate formation at the assayed time point. (A, B) Error bars denote mean ± SD, *N* = 3 biological replicates. The Void^+^ and Aggregate^+^ strains were assayed until *T* = 32 h. The wild-type (WT) strain was assayed until *T* = 26 h. All strains were assayed again at *T* = 42 h if no aggregation/void formation had occurred by the earlier time point. Unpaired two-tailed *t* test comparing measured onset time delay to a mock experiment with no onset timing delay. *, *P* < 0.05; ***, *P* < 0.0005; ns, not significant; #, unable to estimate due to lack of data variance. For samples assayed at *T* = 42 h, onset time was assigned to *T* = 26 h for the purpose of statistical analysis. All strains constitutively expressed *mKO* from the chromosome.

Given that the extracellular proteases Vca0812 and Vca0813 control both void and aggregation onset timing, and their elimination did not fully abrogate either process, other V. cholerae extracellular proteases were candidates for program control. The V. cholerae genome is predicted to encode genes specifying seven additional extracellular proteases, namely, VesA, VesB, VesC, IvaP, TagA, PrtV, and HapA (15, 16, 19–21). We deleted the genes encoding six of these proteases in the Void^+^ strain background. Our attempts to construct an in-frame deletion of *tagA* were unsuccessful, so we engineered a *tagA*::Kan^r^ (Kan^r^, kanamycin resistance) strain. Deletion of *hapA* and *prtV* caused void formation onset timing delays while deletion of *vesA*, *vesB*, *vesC*, or *ivaP*, and transposon insertion into *tagA*, did not (Fig. 4A). Compared to the Void^+^ parent, the Void^+^ strain lacking all four relevant protease-encoding genes (Δ*vca0812* Δ*vca0813* Δ*hapA* Δ*prtV*; designated Δ*4* Void^+^) exhibited severely delayed void formation onset timing by ∼14 h, i.e., longer than that of any mutant lacking any single protease (Fig. 4A). Using the Aggregate^+^ strain as the parent, we showed that HapA and PrtV played analogous roles in delaying aggregate onset formation, and again, simultaneous elimination of all four relevant proteases caused the most severe delay (Fig. 4B). Proper timing, or nearly proper timing in the case of PrtV, was restored following complementation with the respective gene expressed from an ectopic chromosomal locus (Fig. S1B). Again, there were no growth defects (Fig. S3B). We conclude that, in addition to Vca0812 and Vca0813, the HapA and PrtV extracellular proteases contribute to the control of void and aggregate onset timing. We also tested whether elimination of these four proteases delayed aggregation in an otherwise wild-type (WT) strain (i.e., in a strain harboring an intact QS circuit, that produces VPS, and that possesses a functional flagella). Indeed, WT V. cholerae in which the *vca0812*, *vca0813*, *hapA*, and *prtV* genes had been deleted exhibited a severe onset delay in aggregation (Fig. 4B).

### Proteolytic activity is required for control of void and aggregation onset timing.

**FIG 5:**
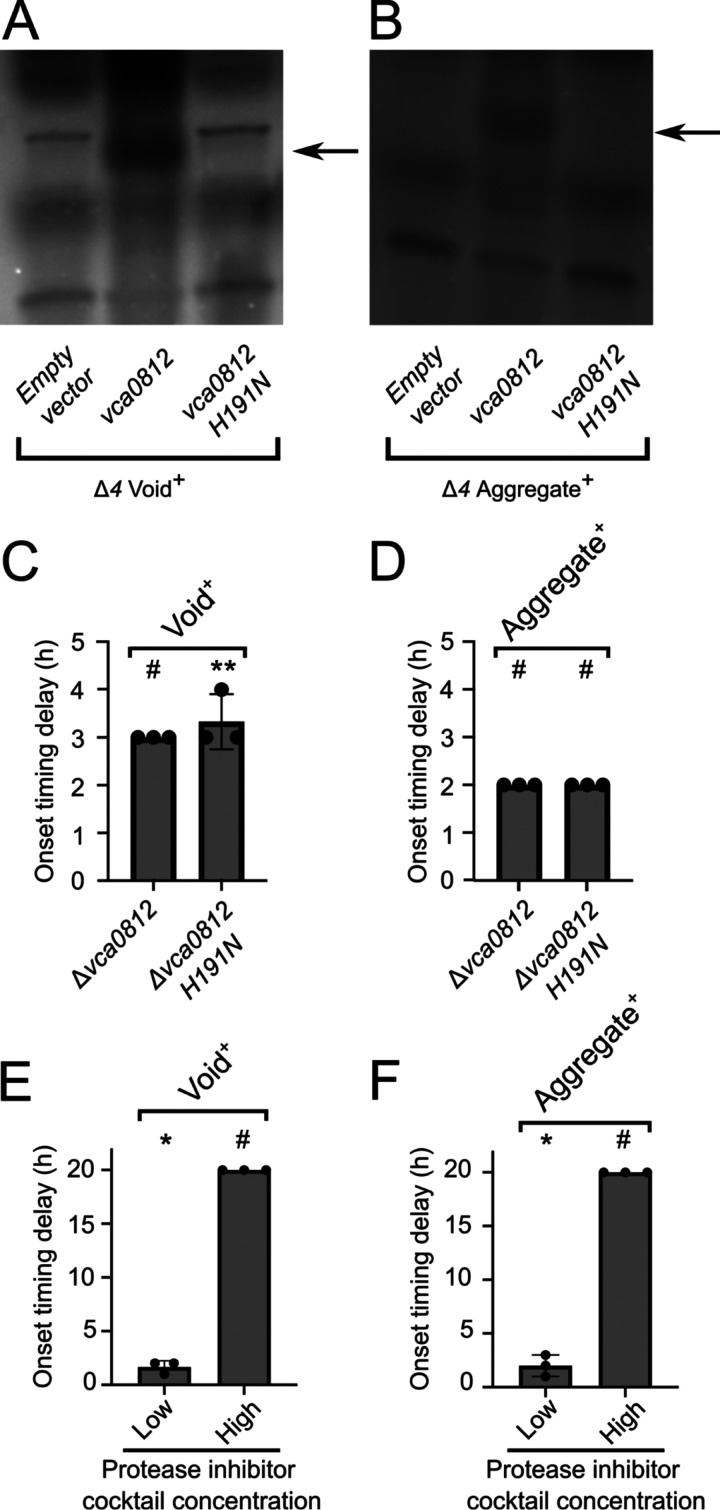
Vca0812 proteolytic activity is required for proper V. cholerae void and aggregation onset timing. (A) Zymographic analyses of proteolytic activity in cell-free culture fluids from the Δ*4* Void^+^ strain, expressing the indicated genes from the pEVS plasmid, under the control of the P_bad_ promoter. (B) As in panel A, for the Δ*4* Aggregate^+^ strain. (A, B) Arrows indicate the regions corresponding to Vca0812 activity. Gels were cropped to show the relevant regions and the lookup table was inverted. The presence of a band indicates proteolytic activity. (C) Quantitation of void formation onset timing delay for the designated strains relative to that of the Void^+^ strain. (D) Quantitation of aggregation onset timing delay for the designated strains relative to that of the Aggregate^+^ strain. (E) Quantitation of void formation onset timing delay for the Void^+^ strain to which a low or high concentration of a protease inhibitor cocktail was administered at *T* = 0 h, relative to the onset time of the Void^+^ strain. (F) Quantitation of aggregation onset timing delay of the Aggregate^+^ strain to which a low or high concentration of a protease inhibitor cocktail was added at *T* = 0 h, relative to the onset time of the Aggregate^+^ strain. (E, F) Samples were assayed from *T* = 22 h until *T* = 26 h, and then again at *T* = 42 h if no void formation or aggregation had occurred by *T* = 26 h. The low protease cocktail concentration was 1 tablet of Roche cOmplete protease inhibitor cocktail per 100 mL, and the high protease cocktail concentration was 1 tablet per 20 mL. (C to F) All strains constitutively expressed *mKO* from the chromosome. Unpaired two-tailed *t* test comparing measured onset time delay to a mock experiment with no onset timing delay. *, *P* < 0.05; **, *P* < 0.005; ns, not significant; #, unable to estimate due to lack of data variance. For samples assayed at *T* = 42 h, onset time was assigned at *T* = 26 h for the purpose of statistical analysis.

A model capturing our above findings is that Vca0812, Vca0813, PrtV, and/or HapA proteolytic activity is required for void formation and aggregation to commence with WT timing. We predict that these proteases cleave a substrate. Cleavage either converts a precursor into an active form required for void formation and aggregation or, alternatively, cleavage inactivates an inhibitor that suppresses void formation and aggregation. To test the necessity for proteolytic activity, we employed a mutant defective in proteolytic activity and, independently, we chemically perturbed protease activity. Regarding the catalytic mutant, we focused on Vca0812 and Vca0813 because they exert the strongest effects on void formation and aggregation onset timing (see Fig. 3 and 4). Crystal structures of Vca0812 and Vca0813 do not exist, so we used I-TASSER to thread Vca0812 and Vca0813 onto homologous proteins from the Protein Data Bank (22). This strategy yielded a predicted catalytic triad for Vc0812, i.e., His191, Asp236, and Ser319. Parallel efforts with Vca0813 were unsuccessful. To test the requirement for catalysis in void formation, we introduced a plasmid harboring either Vca0812 or Vca0812 H191N driven by the P_bad_ promoter into the Δ*4* Void^+^ strain. Zymography showed a proteolytically active band in the strain carrying *vca0812* that is absent from the strain carrying *vca0812 H191N* and the strain harboring the empty vector control (Fig. 5A). Analogous results were obtained with the Δ*4* Aggregate^+^ strain (Fig. 5B). Western blotting verified that the amounts of Vca0812 and Vca0812 H191N were similar in the Δ*4* Void^+^ (Fig. S5A) and Δ*4* Aggregate^+^ (Fig. S5B) strains, although the mutant protein exhibited somewhat higher susceptibility to degradation in the Δ*4* Aggregate^+^ strain (Fig. S5B). We conclude that Vca0812 has proteolytic activity and that Vca0812 H191N is defective for catalysis. Void formation onset timing in the *vca0812 H191N* Void^+^ strain was similar to that of the Δ*vca0812* Void^+^ strain, i.e., they had ∼3- and ∼3.3-h delays, respectively, relative to onset for the parent Void^+^ strain (Fig. 5C). In the case of aggregation, the timing delay of the *vca0812 H191N* Aggregate^+^ strain was similar to that of the Δ*vca0812* Aggregate^+^ strain (2-h delay for both; see Fig. 5D). We conclude that Vca0812 proteolytic activity is required for proper onset timing of void formation and aggregation.

10.1128/mBio.01518-21.5 FIG S5 Vca0812 and Vca0812 H191N proteins are produced at similar levels. Western blots showing the levels of the Vca0812-FLAG and Vca0812 H191N-FLAG proteins produced by the Δ*4* Void^+^ strain (A) and the Δ*4* Aggregate^+^ strain (B). The black arrows designate the positions of the Vca0812-FLAG and Vca0812 H191N-FLAG proteins. Download FIG S5, TIF file, 2.2 MB. Copyright © 2021 Jemielita et al. 2021 Jemielita et al. https://creativecommons.org/licenses/by/4.0/ This content is distributed under the terms of the Creative Commons Attribution 4.0 International license.

In an independent test for the requirement for proteolytic activity in void formation and aggregation, we used a broad-spectrum protease inhibitor cocktail (see Materials and Methods). We added it to the Void^+^ and Aggregate^+^ strains at time (*T*) = 0 h. Low and high inhibitor concentrations drove ∼2- and >4-h delays, respectively, in void formation onset timing in the Void^+^ strain (Fig. 5E). In the Aggregate^+^ strain, ∼1.6- and >4-h aggregation delays occurred following, respectively, low- and high-concentration inhibitor treatments (Fig. 5F). Only modest growth rate reductions occurred when the protease inhibitor was present (Fig. S3C), suggesting that changes in growth rate are not responsible for differences in void formation onset timing. We conclude that proteolytic activity contributes to the proper onset timing of void and aggregate formation.

### Extracellular proteases can be shared during V. cholerae void and aggregate formation.

**FIG 6:**
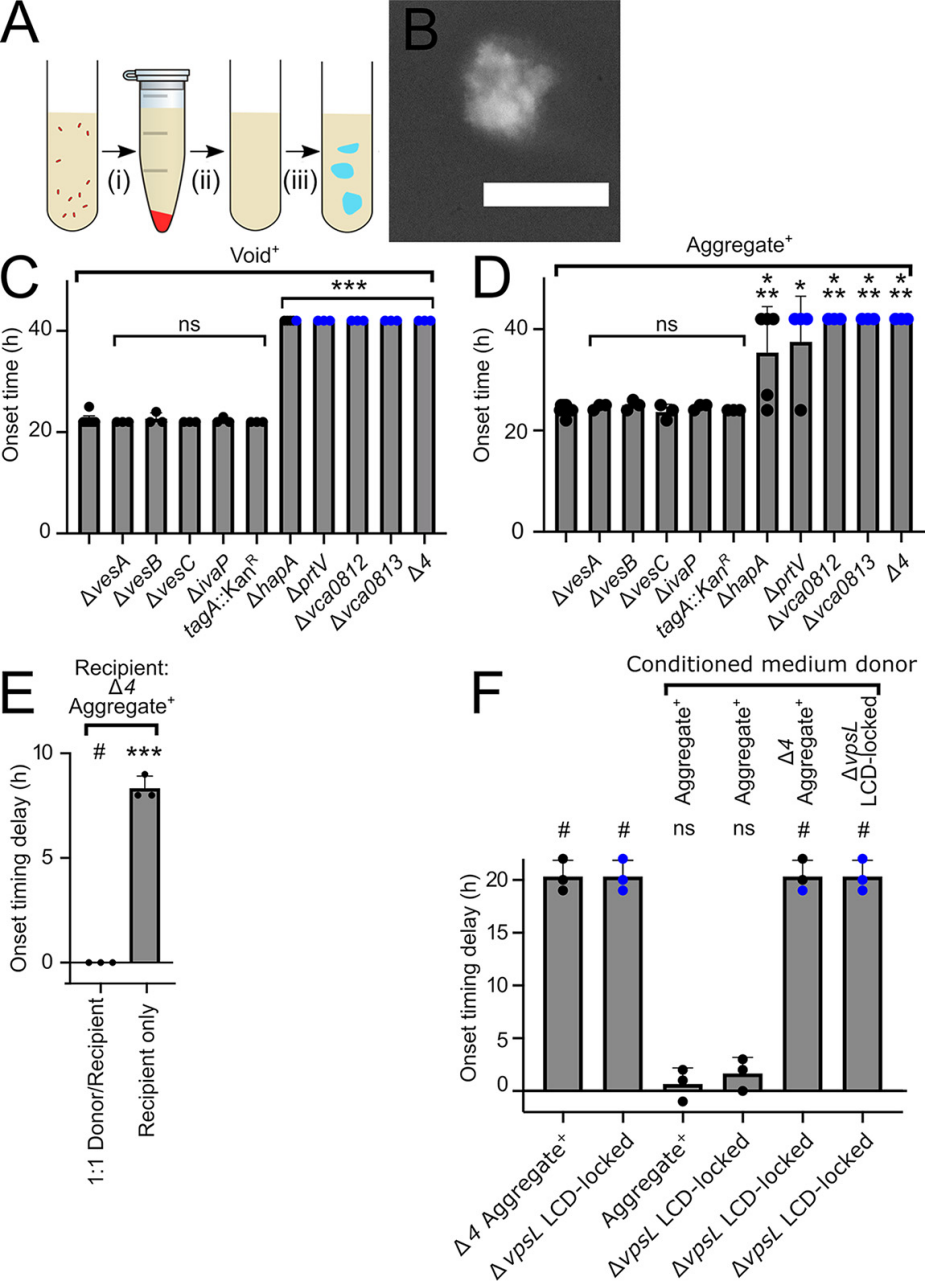
Cell-free conditioned medium forms voids, and the void-promoting activity can be cross-fed to recipient cells. (A) Protocol for cell-free void isolation. Cells were removed via centrifugation (i), and the conditioned medium was filter sterilized (ii). This preparation was further incubated, and cell-free voids formed (iii). (B) Image of a cell-free void (protocol step iii in panel A). Gray, India ink counterstain (inverted lookup table). Bar, 100 μm. Magnification, ×63. (C) Onset time of cell-free void formation for the designated strains in the Void^+^ strain background. (D) Onset time of cell-free void formation for the designated strains in the Aggregate^+^ strain background. (C, D) Error bars show mean ± SD, *N* ≥ 3 biological replicates. Conditioned medium generated at *T* = 18 h. Strains were assayed from *T* = 22 h until *T* = 26 h, and then again at *T* = 42 h if no cell-free void formation had occurred by *T* = 26 h. (E) Aggregation onset delay for a coculture of the Aggregate^+^ (donor) and Δ*4* Aggregate^+^ (recipient) strain and the Δ*4* Aggregate^+^ strain alone relative to the onset timing of the Aggregate^+^ strain. The Aggregate^+^ and the Δ*4* Aggregate^+^ strain constitutively expressed *mKate2* and *mKO*, respectively, from the chromosome. Error bars show mean ± SD, *N* = 3 biological replicates. (F) Aggregation onset delay for the designated strains that had been supplied the indicated conditioned medium at *T* = 18 h relative to the onset timing of the Aggregate^+^ strain to which no conditioned medium was supplied. Samples were assayed from *T* = 22 h until *T* = 26 h, and then again at *T* = 42 h if no void formation or aggregation had occurred by *T* = 26 h. (C, D, F) Blue circles indicate samples that did not exhibit void formation or aggregation at the assayed time point. Strains in panels B, C, D, F constitutively expressed *mKO* from the chromosome. (C, D) Unpaired two-tailed *t* test comparing measured onset time to the Void^+^ (C) or Aggregate^+^ (D) control. *, *P* < 0.05; ***, *P* < 0.0005; ns, not significant. For samples assayed at T = 42 h, onset time was assigned at *T* = 26 h for the purpose of statistical analysis. (E, F) Unpaired two-tailed *t* test comparing measured onset time delay to a mock experiment with no onset timing delay. ***, *P* < 0.0005; ns, not significant; #, unable to estimate due to lack of data variance. For samples assayed at *T* = 42 h, onset time was assigned at *T* = 26 h for the purpose of statistical analysis.

Our finding that extracellular proteases are involved in void and aggregate formation suggested the possibility that, in the absence of cells, components in cell-free fluids might be sufficient to drive the process. To test this idea, immediately prior to void formation onset, we removed the Aggregate^+^ cells from their growth medium and allowed the conditioned medium to continue to incubate (Fig. 6A). Structures resembling voids (Fig. 2B and C) spontaneously formed (Fig. 6B). We call these structures “cell-free voids.” When such conditioned medium was prepared from Void^+^ strains from which we had deleted *vca0812*, *vca0813*, *prtV*, *hapA*, or all four of these genes (Δ*4*), the preparations were incapable (Δ*vca0812*, Δ*vca0813*, Δ*prtV*, and Δ*4*) or severely defective (Δ*hapA*) in promoting cell-free void formation (Fig. 6C). In contrast, conditioned medium prepared from Void^+^ strains from which we had deleted *vesA*, *vesB*, *vesC*, or *ivaP*, or inactivated *tagA* by transposon insertion, (i.e., genes encoding the proteases for which we found no role in void and aggregate formation) formed cell-free voids with the same onset time as those formed in the conditioned medium from the parent Void^+^ strain (Fig. 6C). Parallel results were obtained for the Aggregate^+^ strain, with one difference, namely, that conditioned medium from strains lacking *hapA* or *prtV* exhibited heterogeneity in onset timing delay (Fig. 6D). Combined, these data argue that the four identified extracellular proteases function as factors that promote cell-free void formation.

Bacterial exoproducts are often shared among cells within communities (23). To explore whether this is the case for the proteases controlling void and aggregation formation onset timing, we cocultured protease-producing strains with strains lacking the proteases and assayed whether the nonproducing strains regained WT aggregation timing. Specifically, we combined equal amounts of two strains at *T* = 0 h. The donor strain was the Aggregate^+^ strain, which carries all the extracellular proteases required for proper void formation and aggregation timing. The recipient strain was the Aggregate^+^ strain lacking the four proteases that control void formation and aggregation onset timing (Δ*4* Aggregate^+^ strain). The donor and recipient strain were labeled with *mKate2* and *mKO*, respectively, which enabled us to track the cells of each strain. Coculture allowed the defective recipient strain to regain the shorter, WT aggregation onset timing (Fig. 6E). Importantly, the aggregates that formed in the coculture contained both Aggregate^+^ donor and protease-deficient recipient cells (Video S1). Coculture of the Aggregate^+^ donor with recipient strains lacking only one of the key proteases (i.e., Δ*vca0812*, Δ*vca0813*, or Δ*prtV*) also shortened the duration of the recipients’ delay in aggregation onset timing (Fig. S6). We were unable to resolve timing differences in cocultures of the Aggregate^+^ donor strain with a recipient strain lacking *hapA*. We conclude that the activities of the proteases *vca0812*, *vca0813*, and *prtV*, and/or the components that are processed by them, can be shared among cells.

10.1128/mBio.01518-21.6 FIG S6 Coculture of protease-deficient strains with the Aggregate^+^ strain restored aggregation onset timing. Quantitation of aggregation onset timing delay of the designated strains relative to that of the Aggregate^+^ strain. The donor strain is the Aggregate^+^ strain. Equal amounts of the donor and recipient were combined at *T* = 0 h. Error bars denote mean ± SD, *N* = 3 biological replicates. Donor and recipient strains expressed *mKate2* and *mKO*, respectively, from the chromosome. Unpaired two-tailed *t* test comparing measured onset time delay to a mock experiment with no onset timing delay. *, *P* < 0.05; **, *P* < 0.005; ns, not significant. Download FIG S6, TIF file, 2.7 MB. Copyright © 2021 Jemielita et al. 2021 Jemielita et al. https://creativecommons.org/licenses/by/4.0/ This content is distributed under the terms of the Creative Commons Attribution 4.0 International license.

10.1128/mBio.01518-21.8 VIDEO S1 Cross-feeding between Aggregate^+^ and Δ*4* Aggregate^+^ strains results in the formation of mixed-strain aggregates. Representative *z*-scan at *T* = 24 h of a culture containing a 1:1 (vol/vol) mixture of the Aggregate^+^ and Δ*4* Aggregate^+^ strains. The Aggregate^+^ and Δ*4* Aggregate^+^ strains, respectively, constitutively expressed *mKate2* (red) and *mKO* (yellow) from the chromosome. Bar, 50 μm. Magnification, ×63. Download Movie S1, AVI file, 2.7 MB. Copyright © 2021 Jemielita et al. 2021 Jemielita et al. https://creativecommons.org/licenses/by/4.0/ This content is distributed under the terms of the Creative Commons Attribution 4.0 International license.

We also examined whether providing the proteases in fluids lacking donor cells could rescue a mutant incapable of aggregation. To do this, we generated cell-free conditioned medium from the Aggregate^+^ strain and the Δ*4* Aggregate^+^ strain. We supplied these preparations to the nonaggregating Δ*vpsL* LCD-locked strain at *T* = 18 h. Conditioned medium from the Aggregate^+^ strain elicited aggregation, while conditioned medium from the Δ*4* Aggregate^+^ strain did not (Fig. 6F). We conclude that proteases present in culture fluids are sufficient to restore aggregation to a nonaggregating recipient strain. At present, we do not know whether the shared component is the protease itself, the product of protease digestion, or both.

### Aggregate formation occurs in Vibrio harveyi.

We wondered whether multicellular aggregate formation is specific to V. cholerae or, perhaps, occurs more broadly among vibrios. To preliminarily explore this question, we assayed the bioluminescent marine bacterium Vibrio harveyi for the ability to form aggregates. Both WT and HCD-locked V. harveyi strains formed aggregates similar to those made by V. cholerae (Fig. S7A and B). Similar to V. cholerae, other vibrios produce proteases in the HCD QS state (24, 25). Further work may uncover whether V. harveyi, and perhaps other vibrios, uses a protease-dependent strategy analogous to that of V. cholerae to control the onset timing of multicellular community formation. Finally, while our focus is on marine vibrios, we note that terrestrial bacteria such as Pseudomonas aeruginosa also form aggregates in liquid (26, 27).

10.1128/mBio.01518-21.7 FIG S7 Aggregation occurs *in* Vibrio harveyi. Representative cross-sectional images of wild-type (WT) V. harveyi (A) and a high cell density (HCD)-locked V. harveyi strain (B) at *T* = 22 h visualized using the fluorescent stain SYTO-9. Samples representative of *N* = 3 biological replicates. Bar, 500 μm. Download FIG S7, TIF file, 2.2 MB. Copyright © 2021 Jemielita et al. 2021 Jemielita et al. https://creativecommons.org/licenses/by/4.0/ This content is distributed under the terms of the Creative Commons Attribution 4.0 International license.

## DISCUSSION

Aggregative community formation is a program of V. cholerae multicellularity that occurs rapidly during the stationary phase and when cells are in the HCD QS state (5). We propose that the aggregation program can be divided into two subprograms. One is responsible for void formation, and the other, mediated by the flagellar machinery, facilitates cell entry into voids. Here, we demonstrate that four extracellular proteases, Vca0812, Vca0813, HapA, and PrtV, control the onset timing of void formation and thus aggregation. V. cholerae is predicted to possess nine total extracellular proteases (15, 16, 19–21). The four proteases for which we identified roles are reported to be the most active extracellular proteases during the stationary phase (16). Moreover, QS activates the expression of the genes encoding them at HCD (16), consistent with the timing we discovered for aggregation onset (Fig. 3 and 4). Beyond similar regulation by QS, to our knowledge, these four proteases do not possess shared features that would readily explain their roles in void formation and aggregation. Roles in controlling surface biofilm formation have been ascribed to HapA and PrtV (28, 29), but to our knowledge, no such roles have previously been assigned to Vca0812 or Vca0813.

As a test case for the involvement of these proteases, we demonstrated that Vca0812 proteolytic activity is required for proper void formation and aggregation program onset timing. While untested, we anticipate that the other three proteases likewise harbor proteolytic capability that contribute to program timing. We note that these proteases could additionally promote void formation and aggregation in a proteolysis-independent manner, for example, by acting as adhesins needed to assemble voids. The proteases are shared among community members as demonstrated by our finding that the conditioned medium from the protease proficient Aggregate^+^ strain can elicit aggregation in the normally nonaggregating Δ*vpsL* LCD-locked strain (Fig. 6F). Hundreds of genes are regulated differently in the Δ*vpsL* LCD-locked strain and the Aggregate^+^ strain, since the entire QS regulon is in the LCD mode in the former strain and in the HCD mode in the latter strain. Thus, apparently, the crucial QS role in void and aggregate formation onset timing is the proper regulation of production of these extracellular proteases and/or regulation of their substrates. We propose that the identified extracellular proteases act on a target substrate(s) and cleave it into a product that fosters void formation and aggregation. Alternatively, cleavage could inactivate an inhibitor that suppresses void formation and aggregation. Substrate identification may be achieved by exploiting the catalytically defective Vca0812 H191N mutant, followed by substrate trapping, purification, and mass spectrometry analysis (30). Our pilot purification studies indicate that voids are highly insoluble. We achieved initial success in solubilizing void material using guanidine hydrochloride treatment. Further characterization is a focus of our ongoing efforts.

Program control by multiple proteases could yield benefits not achievable via the involvement of only a single protease. For example, redundancy could facilitate program fidelity by buffering against fluctuations in protease abundance or required cofactors (31). Additionally, two defining features of proteolytic regulation are that such processes are rapid relative to translational or transcriptional control and, furthermore, changes are irreversible (32). Proteolysis speed may underpin the rapid formation of aggregates (5), and the irreversibility of proteolytic cleavage could enable program commitment once the collective decision to undergo aggregation has been made (33). Again, having multiple proteases involved could ensure robustness or underpin a bet-hedging strategy (34) that protects aggregation program execution from competitors that can disable some, but not all, of the proteases involved in driving void and aggregate formation. Previously, we proposed that V. cholerae uses the aggregation formation program to rapidly assemble communities during infection and/or to enhance the successful dispersal from the human host back into the marine environment (5). If so, employing multiple, redundant proteases could ensure the reliable launch of the program in an environment as dynamic and complex as the human intestine.

In V. cholerae and other bacteria, proteolysis-dependent adhesion systems exist (28, 35–37). In some cases, protease activity reduces biofilm formation capacity. For example, in P. aeruginosa, the periplasmic protease LapG cleaves the adhesin CdrA, releasing CdrA from the cell surface, the consequence of which is a reduction in biofilm formation (37). In contrast, proteolytic processing can also be essential for biofilm maturation. In V. cholerae, cleavage of RbmA by the extracellular proteases HapA, PrtV, or IvaP is important for the development of the WT biofilm architecture (36). Beyond prokaryotes, the void formation process has parallels to mechanisms for mammalian blood coagulation (38). When the vascular system sustains injury, a series of proteolytic events is initiated that converts fibrinogen, a soluble protein in blood, into fibrin, which rapidly polymerizes to form a clot. Our evidence suggests that void formation may employ an analogous strategy to rapidly form structures in liquid. In the case of void formation, we suspect that the putative protease substrate is extracellular, because cell-free voids form in conditioned medium lacking cells (Fig. 6B to D). This substrate may undergo autoprocessing, since elimination of all four proteases that individually alter onset timing does not fully eliminate void formation and aggregation. Alternatively, another extracellular protease that we did not identify could exist and fulfill this function.

Finally, formation of voids could occur by a phase transition mechanism. Proteolytic degradation of a substrate could convert it into a form that preferentially adheres to itself or to another component present in the cell-free fluids because of, for example, changes in charge or hydrophobicity. Because the void formation process is proteolytically driven, it is likely irreversible, and the concentration of the proteolyzed product should increase over time until it achieves a level that allows the system to lower its free energy by spontaneously demixing. This process is called spinodal decomposition (39). Key features of spinodal decomposition are that the process is driven by local concentration fluctuations and it occurs spontaneously without the need to overcome an energetic barrier, as is the case with, for example, nucleation-driven phase separation. One difference between void formation and aggregation and classical theories of spinodal decomposition is that, in the latter case, demixing is initiated at a characteristic length scale, but further coarsens over time (39). In contrast, aggregates eventually stop enlarging (5). In future work, we aim to quantitatively study void formation to determine whether spinodal decomposition provides an appropriate framework for understanding. In summary, our work demonstrates that extracellular proteases play a key role in controlling the onset of the V. cholerae aggregative community formation program.

## MATERIALS AND METHODS

### Reagents and bacterial cultures.

The parent strain was V. cholerae O1 El Tor biotype C6706str2 (40). When antibiotics were required, they were used at the following concentrations: ampicillin, 100 mg/L; kanamycin, 100 mg/L; polymyxin B, 50 U/L; chloramphenicol, 10 mg/mL; and streptomycin, 500 mg/L. X-Gal (5-bromo-4-chloro-3-indolyl-β-d-galactopyranoside) was used at 50 mg/L. Strains used in this work are listed in Table S2A in the supplemental material.

10.1128/mBio.01518-21.10 TABLE S2 (A) Strains used in this study. (B) Primers used for strain construction. Download Table S2, PDF file, 0.7 MB. Copyright © 2021 Jemielita et al. 2021 Jemielita et al. https://creativecommons.org/licenses/by/4.0/ This content is distributed under the terms of the Creative Commons Attribution 4.0 International license.

### Strain construction.

Primers used in this study are listed in Table S2B. We constructed chromosomal alterations in V. cholerae strains primarily using multiplex genome editing by natural transformation (MuGENT) (41), and also using allelic exchange with pKAS32 (42) as needed. Both methods have been previously described (5). The MuGENT method relies on natural transformation and cotransformation with a selectable marker at a neutral locus for in-frame deletions. We used *vc1807* as our neutral locus.

### Transposon mutagenesis screen.

We mutagenized the Δ*flgC* Δ*vpsL* HCD-locked *vc1807*::Cm^r^ (Cm^r^, chloramphenicol resistance) strain with Tn*5* as previously described (43). Mutants were selected on Luria broth (LB) agar containing polymyxin B, kanamycin, and 0.5% glycerol. The addition of glycerol amplified differences in colony morphologies (44). Plates were incubated at 30°C for 24 h and subsequently transferred to room temperature for 2 days. Exconjugants exhibiting reductions in opacity were isolated. Mutant strains were grown under aggregate-forming-conditions and screened for the loss of void formation at *T* = 22 h. Transposon insertion sites were determined using arbitrary PCR (45) and subsequently validated with primers specific to the identified loci. To generate the images shown in Fig. S2, 1 mL of culture was grown overnight in LB at 37°C with shaking (250 rpm), and diluted to an optical density (OD) of ∼0.5 in fresh LB medium. A 1-μL aliquot was spotted onto LB plus 0.5% glycerol plates and allowed to incubate as described above. Colonies were imaged using a smartphone with ambient light.

### Aggregate formation.

We used previously described aggregate-forming conditions (5). In brief, V. cholerae strains were grown overnight at 30°C in the outer ring of a rolling drum (model no. M1053-4004, 1 Hz; New Brunswick) in Luria broth (catalog no. BP1426-2; Fisher BioReagents) supplemented with 10 mM Ca^2+^. To image aggregates, a 150-μL sample was placed into a well of a 96-well microtiter dish (no. 1.5 coverslip, catalog no. P96G-1.5-5 F; MatTek). To image voids, a 10-μL sample was gently mixed with 5 μL of India ink (catalog no. 44201; Higgins) on a 60-mm by 44-mm coverslip (no. 1.5), and then a 44-mm by 44-mm coverslip (no. 1.5) was placed on top. Brightfield imaging was used to visualize and quantify void formation. Fluorescence imaging, as previously described (5), was used to visualize aggregate formation. V. harveyi samples were grown and aggregates assessed under identical conditions to those used for V. cholerae except that Luria marine (LM) medium supplemented with 10 mM Ca^2+^ was used as the growth medium.

### Conditioned medium preparation.

Strains were grown (10 mL total volume in 5 technical replicates) under aggregate-forming conditions until *T* = 18 h and then pooled. Cultures were subjected to centrifugation for 30 min at 10,000 rpm on in a Sorvall RC 5B Plus centrifuge and then filter sterilized (pore size 0.22 μm, catalog no. SLGP033R; MilliporeSigma). A 2-mL aliquot of conditioned medium was placed into a test tube and returned to a rolling drum at 30°C until the designated time point. Samples were visualized as described above.

### Proteolytic zymography activity.

Strains were grown overnight in LB medium with 0.1% arabinose at 37°C with shaking (250 rpm). The optical density at 600 nm (OD_600_) values of cultures were measured, and the cells were removed from the suspensions by centrifugation at 13,000 rpm for 10 min. The conditioned media were retained, sterilized by passage through a 0.22-μm filter (MilliporeSigma), the amounts normalized to the culture OD_600_ using water, and then concentrated ∼40-fold by passage over spin columns (10-kDa molecular cutoff, catalog no. UFC901024 and UFC501096; MilliporeSigma). Proteins present in a 2-μL aliquot of each preparation were separated on a 10% SDS gel containing 0.1% gelatin (catalog no. ZY00105BOX; Thermo Fisher) under nonreducing, nondenaturing conditions. The gel was washed twice with water for 5 min, followed by incubation with renaturation buffer (catalog no. LC2670; Thermo Fisher) for 21 h at 30°C. The gel was stained with Coomassie brilliant blue R-250 (catalog no. 1610436; Bio-Rad) and imaged using transillumination. The lookup table was inverted using ImageJ (NIH). Thus, the presence of a band in the figure represents protease activity in that region of the gel.

### Proteolysis inhibition.

Chemical inhibition of proteolytic activity was accomplished by dissolving a protease inhibitor cocktail tablet (cOmplete, mini, EDTA free, catalog no. 04 693 159 001; Roche) into either 20 mL (high protease inhibitor concentration) or 100 mL (low protease inhibitor concentration) of LB medium containing 10 mM Ca^2+^. This medium was used to grow cultures under aggregate-forming conditions.

### Immunoblotting.

Conditioned media were obtained as described above. We combined 1 μL of these preparations with 4× SDS-PAGE buffer, boiled the samples for 20 min, and separated them on 4% to 20% Mini-Protein TGX gels (catalog no. 4561096; Bio-Rad) at 100 V until the dye front reached the bottom of the gel (∼2 h). Proteins were transferred to polyvinylidene difluoride membranes (catalog no. 1620174; Bio-Rad) for 1 h at 4°C and 100 V. Membranes were incubated at room temperature for 40 min with a 1:5,000 dilution of monoclonal anti-FLAG-peroxidase antibody in phosphate-buffered saline with Tween 20 (PBST) supplemented with 5% milk (catalog no. A8592; Sigma). The membranes were subsequently washed another 5 times with PBST. FLAG epitope-tagged protein levels were visualized using the Amersham ECL Western blotting detection reagent (catalog no. GERPN2209; GE Healthcare). In a parallel gel, 10-μL aliquots of the samples were separated on 4% to 20% stain-free TGX gels (catalog no. 4568095; Bio-Rad) and assessed for total protein following the manufacturer’s recommendations. In all cases, samples were imaged (ImageQuant LAS 4000; GE), and protein levels were quantified using ImageJ.

### Microscopy and image analysis.

Microscopy and image analyses were performed as previously described (5). In brief, we used a Leica SP-8 point scanning confocal microscope equipped with a white-light laser for all confocal imaging. Samples were imaged using either a 10× air or 63× water objective. All features of aggregation and void formation were assessed using the 10× air objective. To determine onset timing of aggregation and void formation, 1,550-μm by 1,550-μm regions were imaged every 1 h, beginning well before any perceivable void formation or aggregation had occurred. Samples were visually scored for the presence or absence of voids or aggregates. Onset timing for Void^+^ and Aggregate^+^ strains were assessed using the void formation and aggregation formation protocols, respectively. To quantify the sizes of voids and aggregates, samples were prepared using the void formation protocol, and three nonoverlapping 4,650-μm by 4,650-μm regions were imaged using the tile scanning module. An intensity-based threshold segmentation on the brightfield channel was used to identify the extent of voids and aggregates across these regions. To determine the fractional occupancy of cells, an intensity-based segmentation was subsequently employed using the relevant fluorescent channel. All image analyses were performed in MATLAB using custom software (https://github.com/jemielim/aggregation). To visualize void formation and aggregation in strains lacking fluorescent reporters, SYTO-9 (final concentration 2.2 μM, catalog no. S34854; Thermo Fisher) was used. Cultures were first aliquoted into wells of microtiter dishes, and then dye was added and samples were gently mixed.

### Growth rate analyses.

Strains were grown as described above, and 150-μL aliquots were added to wells of 96-well clear flat-bottomed polystyrene tissue culture (TC)-treated microplates (catalog no. 3598; Corning). Plates were incubated with shaking at 30°C in a Synergy Neo2 multimode reader (BioTek). OD_600_ was measured every 20 min for 16 h. Growth curves were normalized to the time when the cultures entered the exponential phase, which we accomplished by shifting curves by the time at which the OD_600_ exceeded twice the minimum measured values.
