# Supplementary figures and images for: Secreted Proteases Control the Timing of Aggregative Community Formation in Vibrio cholerae
Source: mBio. 2021 Nov 23;12(6):e01518-21. doi: 10.1128/mBio.01518-21 (PMC8609355; doi:10.1128/mBio.01518-21)

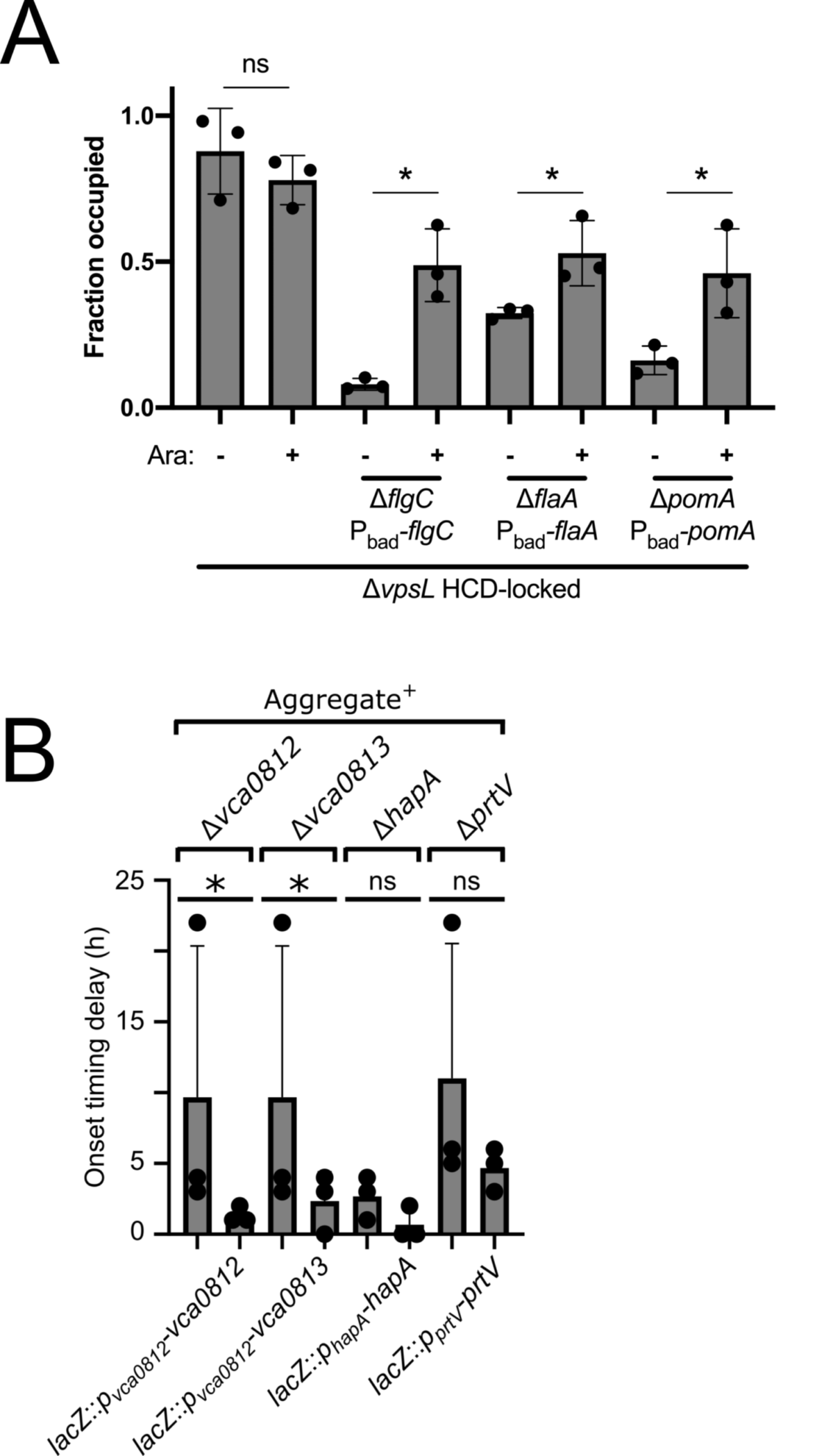

Supplement: FIG S1 [file mbio.01518-21-sf001.tif]

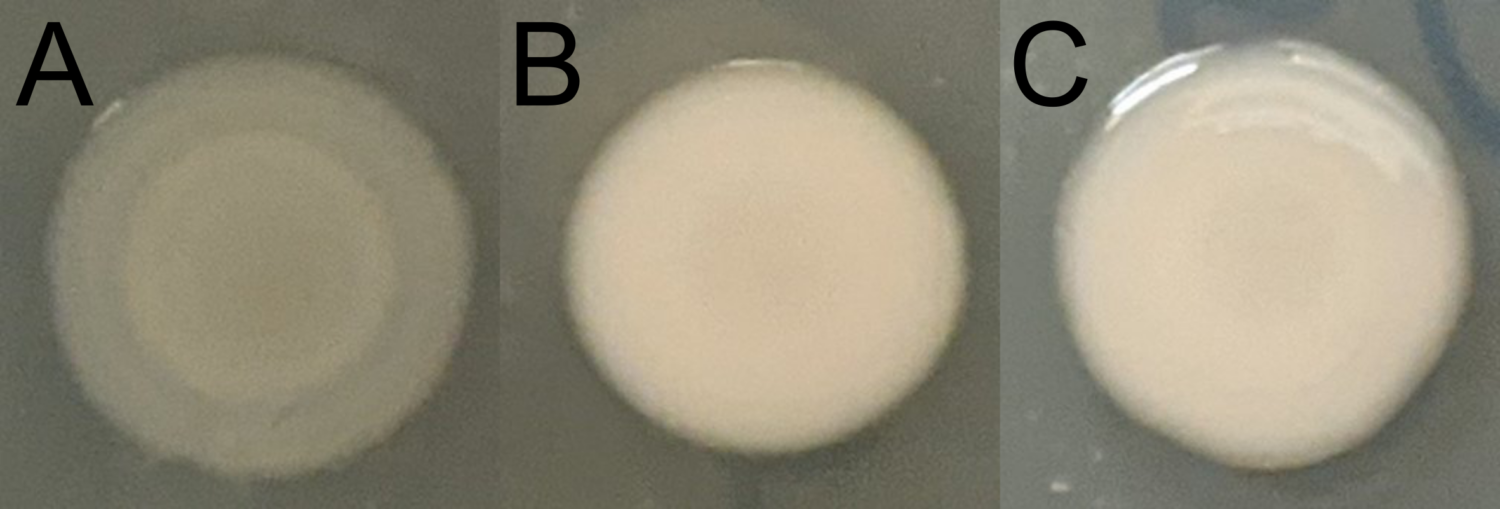

Supplement: FIG S2 [file mbio.01518-21-sf002.tif]

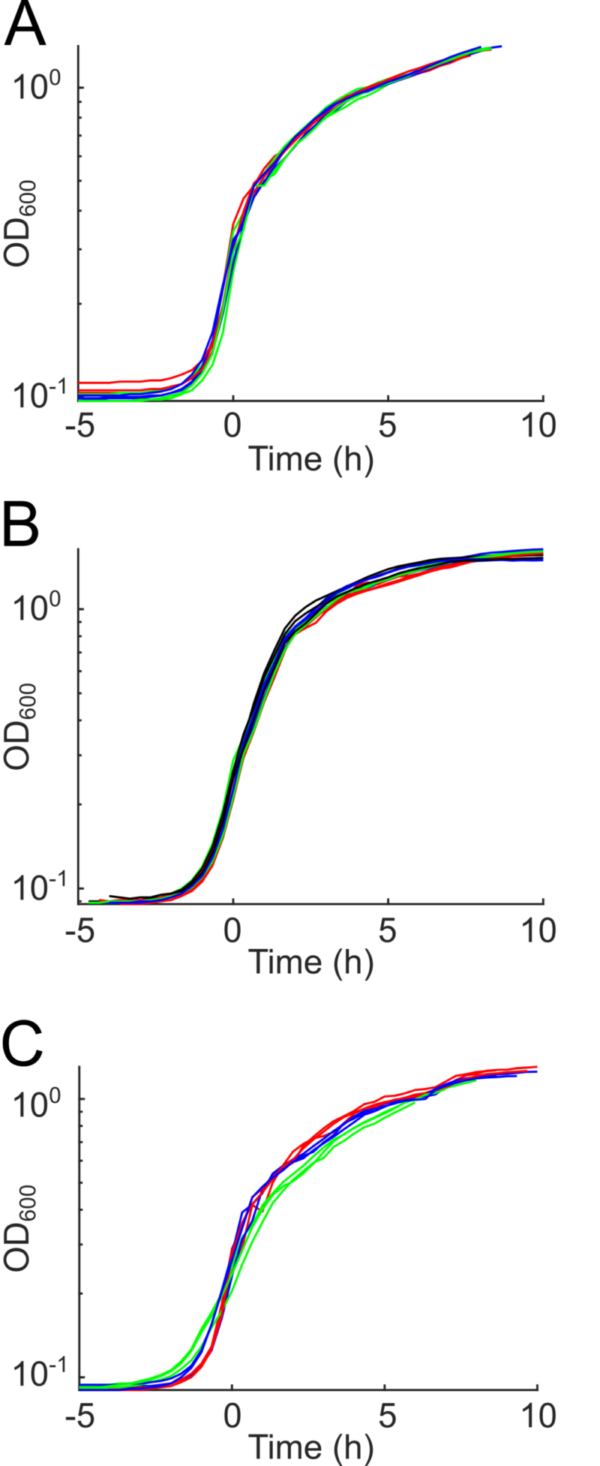

Supplement: FIG S3 [file mbio.01518-21-sf003.tif]

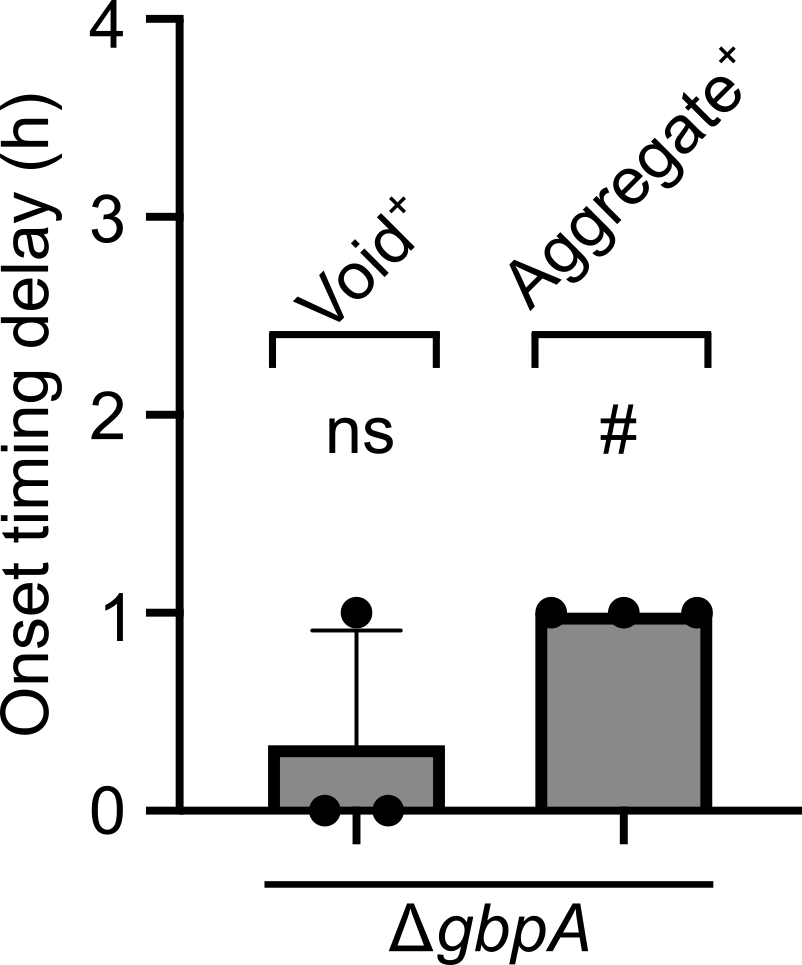

Supplement: FIG S4 [file mbio.01518-21-sf004.tif]

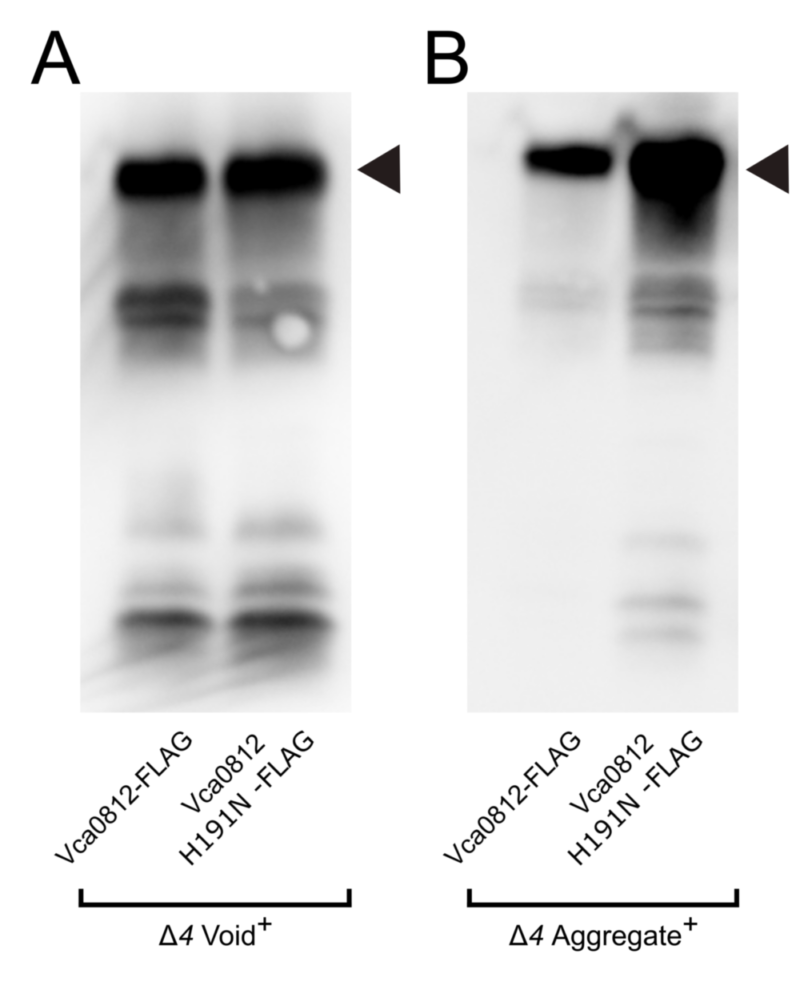

Supplement: FIG S5 [file mbio.01518-21-sf005.tif]

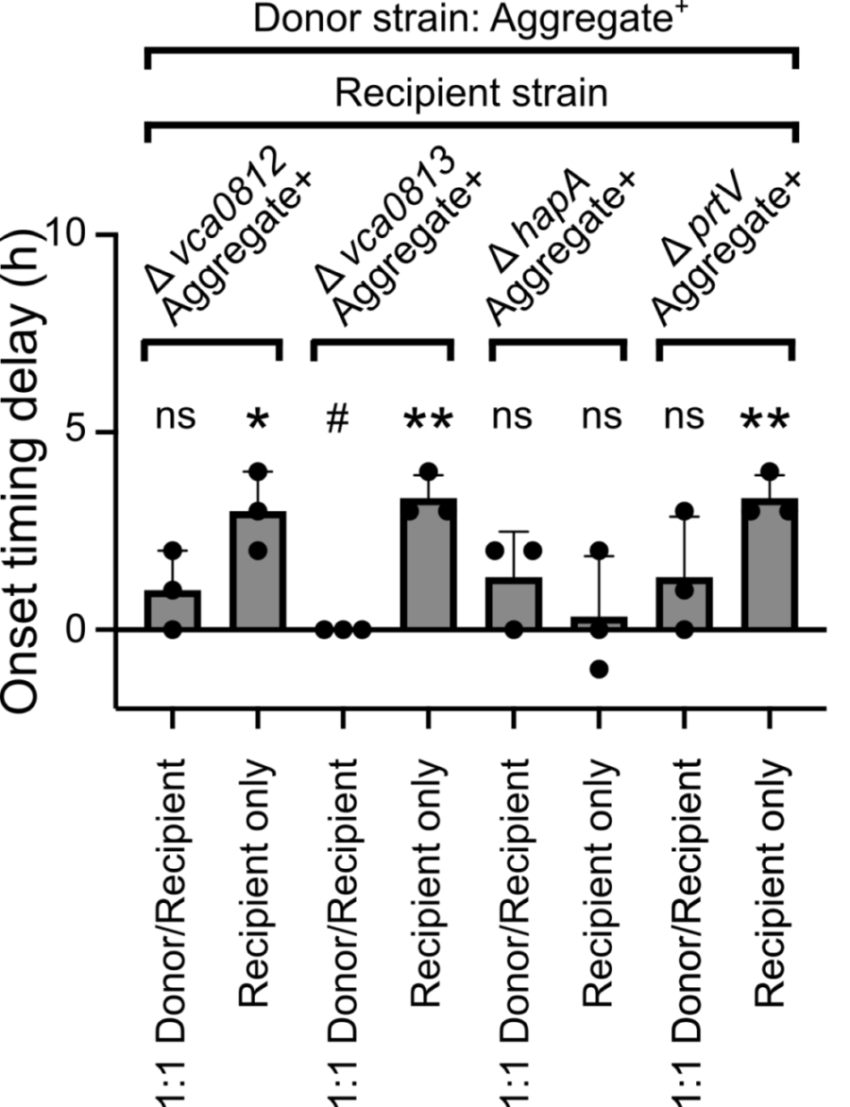

Supplement: FIG S6 [file mbio.01518-21-sf006.tif]

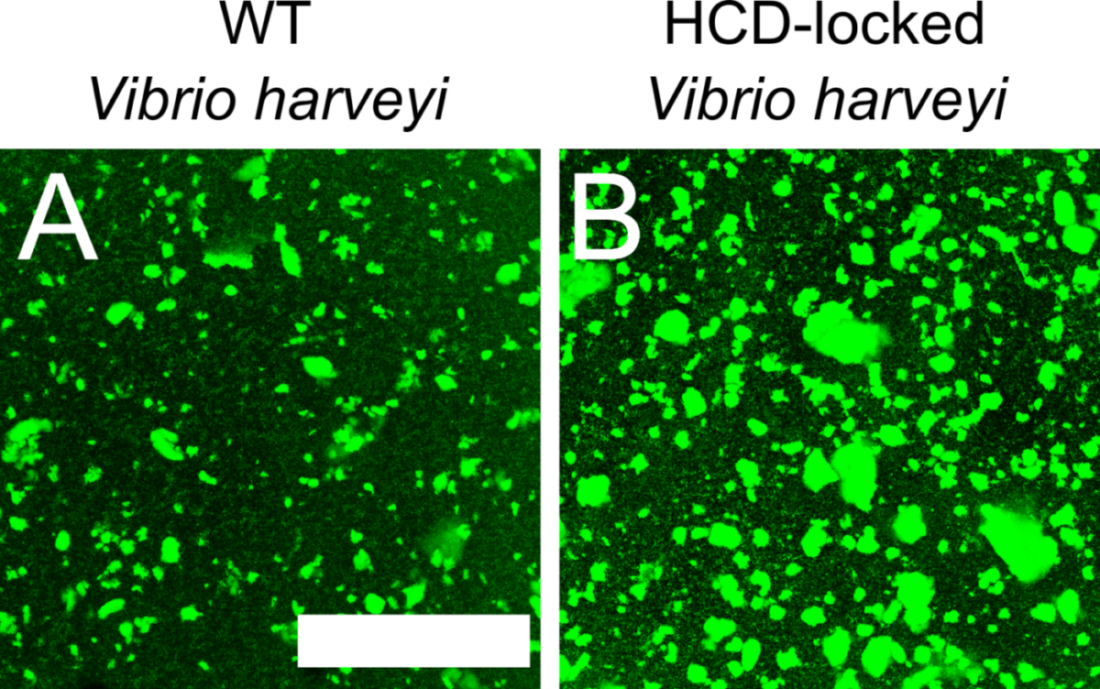

Supplement: FIG S7 [file mbio.01518-21-sf007.tif]
