# Supplementary material for: Secreted Proteases Control the Timing of Aggregative Community Formation in Vibrio cholerae
Source: mBio. 2021 Nov 23;12(6):e01518-21. doi: 10.1128/mBio.01518-21 (PMC8609355; doi:10.1128/mBio.01518-21)
Supplement: TABLE S1 [file mbio.01518-21-st001.pdf]

Supplementary Table 1: Genes identified in screen

Gene locus	Annotation
vc0122	Adenylate cyclase (*cyaA*)
vc0211	Orotate phosphoribosyltransferase (*pyrE*)
vc0276	Phosphoribosylaminoimidazolecarboxamide formyltransferase (*purH*)
vc0384	Sulfite reductase (NADPH) flavoprotein subunit alpha (*cysJ*)
vc0534	RNA polymerase sigma-38 factor (*rpoS*)
vc0576	Stringent starvation protein A (*sspA*)
vc0581	Lipoprotein activator of PBP1A (*lpoA*)
vc0583	LuxR family transcriptional regulator (*hapR*)
vc0768	GMP synthase (*guaA*)
vc0944	Lipoate-protein ligase B (*lipB*)
vc1004	Amidophosphoribosyltransferase (*purF*)
vc1213	Response regulator (*varA*)
vc1491	Dihydroorotate dehydrogenase: pyrimidine metabolism (*pyrD*)
vc1914	Integration host factor B (*ihfB*)
vc2067	Flagellar biosynthesis protein (*flhG*)
vc2200	Flagellar basal-body rod protein (*flgB*)
vc2389	Carbamoyl-phosphate synthase; large subunit (*carA*)
vc2453	Sensor histidine kinase (*varS*)
vc2480	Ribose-5-phosphate isomerase A (*rpiA*)
vc2510	Aspartate carbamoyltransferase (*pyrB*)
vc2544	Fructose-1,6-bisphosphatase (*fbp*)
vc2625	Ribulose-phosphate 3-epimerase
vc2635	Penicillin-binding protein 1A (*mrcA*)
vca0657	Glycerol-3-phosphate dehydrogenase (*glpD*)
vca0812	Leucine aminopeptidase-like protein
