# Supplementary material for: Secreted Proteases Control the Timing of Aggregative Community Formation in Vibrio cholerae
Source: mBio. 2021 Nov 23;12(6):e01518-21. doi: 10.1128/mBio.01518-21 (PMC8609355; doi:10.1128/mBio.01518-21)
Supplement: TABLE S2 [file mbio.01518-21-st002.pdf]

Supplementary Table 2A: Strain list

Strain or plasmid	Relevant features	Reference
V. cholerae		
BB-Vc0616	*flgC*::Tn*5* Δ*vpsL luxO D61A lacZ*::P_{hapR}-*hapR*	(1)
BB-Vc0569	Δ*flgC* Δ*vpsL luxO D61A lacZ*::P_{tac}-*mKO vc1807::Kan*^{R}	This study
BB-Vc0570	Δ*flaA* Δ*vpsL luxO D61A lacZ*::P_{tac}-*mKO vc1807::Kan*^{R}	This study
BB-Vc0571	Δ*pomA* Δ*vpsL luxO D61A lacZ*::P_{tac}-*mKO vc1807::Kan*^{R}	This study
BB-Vc0572	Δ*vpsL luxO D61A lacZ*::P_{tac}-*mKO vc1807::Kan*^{R}	This study
BB-Vc0466	Δ*vca0812* Δ*vpsL luxO D61A lacZ*::P_{tac}-*mKO vc1807::Kan*^{R}	This study
BB-Vc0467	Δ*vca0813* Δ*vpsL luxO D61A lacZ*::P_{tac}-*mKO vc1807::Kan*^{R}	This study
BB-Vc0465	Δ*vca0812* Δ*vca0813* Δ*vpsL luxO D61A lacZ*::P_{tac}-*mKO vc1807::Kan*^{R}	This study
BB-Vc0573	Δ*vca0812* Δ*flgC* Δ*vpsL luxO D61A lacZ*::P_{tac}-*mKO vc1807::Kan*^{R}	This study
BB-Vc0574	Δ*vca0813* Δ*flgC* Δ*vpsL luxO D61A lacZ*::P_{tac}-*mKO vc1807::Kan*^{R}	This study
BB-Vc0575	Δ*vca0812* Δ*vca0813* Δ*flgC* Δ*vpsL luxO D61A lacZ*::P_{tac}-*mKO vc1807::Kan*^{R}	This study
BB-Vc0464	Δ*gbpA* (*vca0811*) Δ*vpsL luxO D61A lacZ*::P_{tac}-*mKO vc1807::Kan*^{R}	This study
BB-Vc0576	Δ*gbpA* (*vca0811*) Δ*flgC* Δ*vpsL luxO D61A lacZ*::P_{tac}-*mKO vc1807::Kan*^{R}	This study
BB-Vc0463	Δ*ivaP* Δ*vpsL luxO D61A lacZ*::P_{tac}-*mKO vc1807::Kan*^{R}	This study
BB-Vc0459	Δ*hapA* Δ*vpsL luxO D61A lacZ*::P_{tac}-*mKO vc1807::Kan*^{R}	This study
BB-Vc0460	Δ*prtV* Δ*vpsL luxO D61A lacZ*::P_{tac}-*mKO vc1807::Kan*^{R}	This study
BB-Vc0577	*tagA*::Kan^R Δ*vpsL luxO D61A lacZ*::P_{tac}-*mKO*	This study
BB-Vc0461	Δ*vesA* Δ*vpsL luxO D61A lacZ*::P_{tac}-*mKO vc1807::Kan*^{R}	This study
BB-Vc0462	Δ*vesB* Δ*vpsL luxO D61A lacZ*::P_{tac}-*mK vc1807::Kan*^{R}	This study
BB-Vc0578	Δ*vesC* Δ*vpsL luxO D61A lacZ*::P_{tac}-*mKO vc1807::Kan*^{R}	This study
BB-Vc0579	Δ*ivaP* Δ*flgC* Δ*vpsL luxO D61A lacZ*::P_{tac}-*mKO vc1807::Kan*^{R}	This study

BB-Vc0580	Δ*hapA* Δ*flgC* Δ*vpsL luxO D61A lacZ*::P_{tac}-*mKO vc1807::Kan*^{R}	This study
BB-Vc0581	Δ*prtV* Δ*flgC* Δ*vpsL luxO D61A lacZ*::P_{tac}-*mKO vc1807::Kan*^{R}	This study
BB-Vc0582	*tagA*::Kan^R Δ*flgC* Δ*vpsL luxO D61A lacZ*::P_{tac}-*mKO vc1807::Cm*^{R}	This study
BB-Vc0583	Δ*vesA* Δ*flgC* Δ*vpsL luxO D61A lacZ*::P_{tac}-*mKO vc1807::Kan*^{R}	This study
BB-Vc0584	Δ*vesB* Δ*flgC* Δ*vpsL luxO D61A lacZ*::P_{tac}-*mKO vc1807::Kan*^{R}	This study
BB-Vc0585	Δ*vesC* Δ*flgC* Δ*vpsL luxO D61A lacZ*::P_{tac}-*mKO vc1807::Kan*^{R}	This study
BB-Vc0314	Δ*prtV* Δ*hapA* Δ*vca0812* Δ*vca0813* (Δ4) Δ*vpsL luxO D61A lacZ*::P_{tac}-*mKO vc1807::Kan*^{R}	This study
BB-Vc0588	Δ*prtV* Δ*hapA* Δ*vca0812* Δ*vca0813* (Δ4) Δ*flgC* Δ*vpsL luxO D61A lacZ*::P_{tac}-*mKO vc1807::Kan*^{R}	This study
BB-Vc0613	Δ*prtV* Δ*hapA* Δ*vca0812* Δ*vca0813* (Δ4) *lacZ*::P_{tac}-*mKO vc1807::Kan*^{R}	This study
BB-Vc0614	*lacZ*::P_{tac}-*mKO vc1807::Kan*^{R}	This study
BB-Vc0594	Δ*vca0812* Δ*vpsL luxO* D61A *lacZ*::$P_{vca0812}$-*vca0812 vc1807::Kan*^{R}	This study
BB-Vc0595	Δ*vca0813* Δ*vpsL luxO* D61A *lacZ*::$P_{vca0813}$-*vca0813 vc1807::Kan*^{R}	This study
BB-Vc0597	Δ*prtV* Δ*vpsL luxO D61A lacZ*::P_{prtV}-*prtV vc1807::Kan*^{R}	This study
BB-Vc0598	Δ*hapA* Δ*vpsL luxO D61A lacZ*::P_{hapA}-*hapA vc1807::Kan*^{R}	This study
BB-Vc0617	*vca0812 H191N* Δ*vpsL luxO D61A lacZ*::P_{tac}-*mKO vc1807::Cm*^{R}	This study
BB-Vc0620	*vca0812 H191N* Δ*flgC* Δ*vpsL luxO D61A lacZ*::P_{tac}-*mKO vc1807::Kan*^{R}	This study
BB-Vc0593	Δ*vpsL* Δ*vca0812* Δ*vca0813* Δ*prtV* Δ*hapA luxO D61A vc1807*::Cm^R pEVS-P_{bad}-empty vector	This study
BB-Vc0606	Δ*vpsL* Δ*vca0812* Δ*vca0813* Δ*prtV* Δ*hapA vc1807*::Cm^R *luxO D61A* pEVS-*vca0812*	This study
BB-Vc0214	Δ*vpsL luxO D61E lacZ::*P_{tac}-*mKO*	(1)
BB-Vc0607	Δ*vpsL* Δ*vca0812* Δ*vca0813* Δ*prtV* Δ*hapA vc1807*::Cm^R	This study

	luxO D61A pEVS-*vca0812 H191N*	
BB-Vc0621	Δ*vpsL* Δ*vca0812* Δ*vca0813* Δ*prtV* Δ*hapA* Δ*flgC* *vc1807*::Cm^R<br>*luxO D61A* pEVS-P_{bad}-empty vector	This study
BB-Vc0622	Δ*vpsL* Δ*vca0812* Δ*vca0813* Δ*prtV* Δ*hapA* Δ*flgC* *vc1807*::Cm^R<br>*luxO D61A* pEVS-*vca0812*	This study
BB-Vc0623	Δ*vpsL* Δ*vca0812* Δ*vca0813* Δ*prtV* Δ*hapA* Δ*flgC* *vc1807*::Cm^R<br>*luxO D61A* pEVS-*vca0812 H191N*	This study
BB-Vc0608	Δ*vpsL* Δ*vca0812* Δ*vca0813* Δ*prtV* Δ*hapA* *vc1807*::Cm^R<br>*luxO D61A* pEVS-*vca0812-FLAG*	This study
BB-Vc0609	Δ*vpsL* Δ*vca0812* Δ*vca0813* Δ*prtV* Δ*hapA* *vc1807*::Cm^R<br>*luxO D61A* pEVS-*vca0812 H191N-FLAG*	This study
BB-Vc0625	Δ*vpsL* Δ*vca0812* Δ*vca0813* Δ*prtV* Δ*hapA* Δ*flgC* *vc1807*::Cm^R<br>*luxO D61A* pEVS-*vca0812-FLAG*	This study
BB-Vc0626	Δ*vpsL* Δ*vca0812* Δ*vca0813* Δ*prtV* Δ*hapA* Δ*flgC* *vc1807*::Cm^R<br>*luxO D61A* pEVS-*vca0812 H191N-FLAG*	This study
BB-Vc0629	Δ*flgC* Δ*vpsL* *luxO D61A* *lacZ*::P_{tac}-*mKO* *vc1807::*P_{bad}-*flgC-Spec*^{R}	This study
BB-Vc0630	Δ*flaA* Δ*vpsL* *luxO D61A* *lacZ*::P_{tac}-*mKO* *vc1807::*P_{bad}-*flaA-Spec*^{R}	This study
BB-Vc0631	Δ*pomA* Δ*vpsL* *luxO D61A* *lacZ*::P_{tac}-*mKO* *vc1807::*P_{bad}-*pomA-Spec*^{R}	This study
<u>V. harveyi</u>		
BB120	WT	(2)
JAF483	BB120 *luxO D47A*-Kan^R	(3)
**<u>Plasmids</u>**	**<u>Genotype</u>**	
BB-Ec366	pEVS-P_{bad}-empty vector	Bassler Lab collection
BB-Ec1169	pEVS-P_{bad}-*vca0812*	This study

BB-Ec1170	pEVS-P_{bad}-*vca0812 H191N*	This study
BB-Ec1172	pEVS *vca0812 H191N-FLAG*	This study
BB-Ec1345	pEVS *vca0812-FLAG*	This study
BB-Ec0362	pKAS32 *lacZ*::P_{tac}-*mKO*	Bassler lab collection

References

1. Jemielita M, Wingreen NS, Bassler BL. 2018. Quorum sensing controls *Vibrio cholerae* multicellular aggregate formation. Elife 7:e42057.
2. Bassler BL, Greenberg EP, Stevens AM. 1997. Cross-species induction of luminescence in the quorum-sensing bacterium *Vibrio harveyi*. J Bacteriol 179:4043-4045.
3. Freeman JA, Bassler BL. 1999. A genetic analysis of the function of LuxO, a two-component response regulator involved in quorum sensing in *Vibrio harveyi*. Mol Microbiol 31:665-677.

Supplementary Table 2B: Primer list

Primer description	Direction	5'→3' sequence
flgC fragment #1	Fwd	CCACACGAATATTTTTTTTGATTTTTGCACCAA
flgC fragment #1	Rev	GGCCATACGTTAACTCCTTATCCTTACATCTATCTACTCCCCTTTAAGCGAC
flgC fragment #2	Fwd	GTCGCTTAAAGGGGAGTAGATAGATGTAAGGATAAGGAGTTAACGTATGGCCGGAGT
flgC fragment #2	Rev	CCTTAAATTAGCTACTGCGAAGCAGTGA
flaA deletion	Fwd	CTGAATGATTTCCATGAGACGGTTCG
flaA deletion	Rev	ACCTAGCTTCGGCTAGGTTTTGTTTTATC
pomA deletion	Fwd	CTTCGCATGCCGTGCTTTCAC
pomA deletion	Rev	CGTTATCAAGGCCGTTCTCTTTGG
vca0812 fragment #1	Fwd	GATAGACGGAATCCGATGACGTTCTATTCG
vca0812 fragment #1	Rev	GTGAAGAATCGTTATTGTTTTTCAGTACTTTTCATCATGATTCCTTCTGAAAGAATGGTT
vca0812 fragment #2	Fwd	TTTCAGAAGGAATCATGATGAAAAGTACTGAAAAACAATAACGATTCTTCACACCC
vca0812 fragment #2	Rev	CATACCTAATGGGCTTTTTCCTGGTCG
vca0813 fragment #1	Fwd	CAATTCAAGCTGGCCCACAAACC
vca0813 fragment #1	Rev	GTGATATCGCTTTTCCCAAGCTACTGGTTCATAAGATTTTCCCTTCTCCGTGG
vca0813 fragment #2	Fwd	CACGGAGAAGGGAAAATCTTATGAACCAGTAGCTTGGGAAAAGCGATATCAC
vca0813 fragment #2	Rev	GCTTTTTTGGCATTGACGATCAAGGC
vca0812-3 deletion	Rev	AAGTTGTTTGGTGATATCGCTTTTCCCAAGGATTCCTTCTGAAAGAATGGTTGTTAAT
vca0812-3 deletion	Fwd	TTATTAACAACCATTCTTTCAGAAGGAATCCTTGGGAAAAGCGATATCACCAAACA
gbpA fragment #1	Fwd	GACACTACAACACAACCCGGAC
gbpA fragment #1	Rev	TAAGAGGGAGGATAACTTAACGTTTATCAGGTTGTTTTTTCATCACAGACTCTTCTT
gbpA fragment #2	Fwd	GAAGAGTCTGTGATGAAAAAACAACCTGATAAACGTTAAGTTATCCTCCCTCTTACACC
gbpA fragment #2	Rev	CATCGGGAACTTGAGATGAGCC
hapA amplification	Fwd	GCTCTTTGTAGGACGTCGTATAGTAAATCG
hapA amplification	Rev	CAATCGGTGAGCAATACTCTGTTTGC
ivaP amplification	Fwd	GTACTTTGTGAGATTCGCTCCACCTC
ivaP amplification	Rev	CTTCCATGTGGTGGGTGTAAGGAAATTG
prtV fragment #1	Fwd	CTTTCACGCCAGAGATAGCGATGTAC
prtV fragment #1	Rev	AGAAGGAAGAATTACAGCGTTTTCATTTTATTTCCTTAATATTTCCTTATTTAAGGTGTG
prtV fragment #2	Fwd	AATATTAAGGAAATAAAATGAAAACGCTGTAATTCTTCCTTCTCCTTCCATGGAT
prtV fragment #2	Rev	CTAACTGTGCTTCGCAGCGAGATAG
vesA fragment #1	Fwd	GCACAAAGAAACCATCGTCACCTTTC
vesA fragment #1	Rev	CTTGATCATAAAGTTCAAATTTTCAGCCATTTGCGCATGCG
vesA fragment #2	Fwd	CAATGAGGTGACGCATGCGCAAATGGCTGAAAATTTGAACTTTATGATCAAGGTGTGT
vesA fragment #2	Rev	CATTCCAGAAGAAGTGTTTGATAAAGCTTCGG
vesB fragment #1	Fwd	GTGAAATACGACAGCTTTGATTCTGCAC
vesB fragment #1	Rev	TTTATCTCGGATAGGTAATCAAGCGGTCTGATGCACAACTCAATCCTTTCATTAATCAG
vesB fragment #2	Fwd	ATTAATGAAAGGATTGAGTTGTGCATCAGACCGCTTGATTACCTATCCGAGA
vesB fragment #2	Rev	CGTGAGCGAATCCACTTCCAAG
vesC fragment #1	Fwd	GAACGCTTGCTGAAAAGTTGTCATCG
vesC fragment #1	Rev	CGAATATCAAGCGGTGGAGGTAGATCGAACTGAAAAGATGTAAAAAGAAGTGTTCCAAC
vesC fragment #2	Fwd	CACTTCTTTTTACATCTTTTCAGTTCGATCTACCTCCACCGCTTGATATTCG

Primer description	Direction	5'→3' sequence
vesC fragment #2	Rev	GCTGGCATATCATCCATAAAGACACTTTCTTG
tagA fragment #1	Fwd	CCGACCATAGATGTCTCTGTGGG
tagA fragment #1	Rev	ATTCCGGGGATCCGTCGACAATACTGGTCGTTACTGGATGTTGCATTCTTTAACAAAAAA
tagA fragment #2	Fwd	TGCAACATCCAGTAACGACCAGTATTGTCGACGGATCCCCGGAA
tagA fragment #2	Rev	ATCAATTATAGGCCTTGATGGCTTCTTGTAGGCTGGAGCTGCTTCAT
tagA fragment #3	Fwd	CGGAGATGAAGCAGCTCCAGCCTACAAGAAGCCATCAAGGCCTATAATTGATG
tagA fragment #3	Rev	ATTGAACATGAATAGTCCTTAATCCTTACTAACTATATGATTACAC
lacZ insertion	Fwd	CAAAGCGACATCCTGCTCAATTGC
lacZ insertion	Rev	GAGAGCACAAGGAGGGTGATTG
lacZ::*vca0812* fragment #1	Rev	GATGATGGGTGTAAGAGGGAGGATAACCCCCACAATAAGCCAGAGAGC
lacZ::*vca0812* fragment #2	Fwd	TTAAGGCTCTCTGGCTTATTGTGGGGGTTATCCTCCCTCTTACACCCATCAT
lacZ::*vca0812* fragment #2	Rev	TTCTTTACTCCTCGGCTTGAGGGATGTTATTGTTTTTCAGTCGCAGTGAGTGAGAC
lacZ::*vca0812* fragment #3	Fwd	CACTCACTGCGACTGAAAAACAATAACATCCCTCAAGCCGAGG
lacZ::*vca0812* fragment #1	Rev	AAAGCCATGACGAATAGTTTGTTCATGCTTAATTACCTCCTAATTGAATTCCTAGG
lacZ::*vca0812* fragment #2	Fwd	GGAATTCAATTAGGAGGTAATTAAGCATGAACAAACTATTCGTCATGGCTTTGA
lacZ::*vca0812* fragment #2	Rev	CTTCTTTACTCCTCGGCTTGAGGGATGCTACTGAGTTGAGGCTTTTAACGC
lacZ::*vca0812* fragment #3	Fwd	TGGCGTTAAAAGCCTCAACTCAGTAGCATCCCTCAAGCCGAGGAGT
lacZ::*hapA* fragment #1	Rev	CGCGGCTTTATTTTCGAAATAGGGCGCCCCACAATAAGCCAGAGAGC
lacZ::*hapA* fragment #2	Fwd	TTAAGGCTCTCTGGCTTATTGTGGGGCGCCCTATTTCGAAAATAAAGCCG
lacZ::*hapA* fragment #2	Rev	ATTCAGAGGACGTTGTATCATTTTCATGCTTAATTACCTCCTAATTGAATTCCTAGGC
lacZ::*hapA* fragment #3	Fwd	GGAATTCAATTAGGAGGTAATTAAGCATGAAAATGATACAACGTCCTCTGAATTGG
lacZ::*prtV* fragment #1	Rev	CTGCCTGACGCCAGAAGCATTGGTGCACCCCACAATAAGCCAGAGAGC
lacZ::*prtV* fragment #2	Fwd	CTTAAGGCTCTCTGGCTTATTGTGGGGTGCACCAATGCTTCTGGCGTCAG
lacZ::*prtV* fragment #2	Rev	TAATAGCGTTTTTTTGATCGTTTTCATGCTTAATTACCTCCTAATTGAATTCCTAGGC
lacZ::*prtV* fragment #3	Fwd	GGAATTCAATTAGGAGGTAATTAAGCATGAAAACGATCAAAAAAACGCTATTAGCTG
vca0812::Kan^R fragment #1	Rev	GAATTGGATTCCGGGGATCCGTCGACCATCATGATTCCTTCTGAAAGAATGGTTGT
vca0812::Kan^R fragment #2	Fwd	CCATTCTTTCAGAAGGAATCATGATGGTCGACGGATCCCCGG
vca0812::Kan^R fragment #2	Rev	CTCCGTGGGTGTGAAGAATCGTTATTGTGTAGGCTGGAGCTGCTTCATCT
vca0812::Kan^R fragment #3	Fwd	CGGAGATGAAGCAGCTCCAGCCTACACAATAACGATTCTTCACACCCACGG
vca0812 H191N	Fwd	CACCAATAATAACTGTGTCAGCACAGC
vca0812 H191N	Rev	GCTGTGCTGACACAGTTATTATTGGTG
plasmid_vca0812_nf_for	Fwd	TTCTCCGCATTTCCAGTGGAGGATATGGCATGATGAAAAGTAAAATAGTGACCGCCACA
plasmid_vca0812_nf_rev	Rev	TGTGGCGGTCACTATTTTACTTTTCATCATGCCATATCCTCCACTGGAAATGCGGAGAA
lap_pvca0812_nf_for	Fwd	CTGGGGTCTCACTCACTGCGACTGAAAAACAATAGTAAGCAACAACGTCAAGCTGATTG
lap_vca0812_nf_rev	Rev	CAATCAGCTTGACGTTGTTGCTTACTATTGTTTTTCAGTCGCAGTGAGTGAGACCCCAG
plasmid_vca0812_flag_for	Fwd	TTCTCCGCATTTCCAGTGGAGGATATGGCATGATGAAAAGTAAAATAGTGACCGCCACA

Primer description	Direction	5’→3’ sequence
plasmid_vca0812_flag_rev	Rev	TGTGGCGGTCACTATTTTACTTTTCATCATGCCATATCCTCCACTGGAAATGCGGAGAA
vca0812_plasmid_flag_for	Fwd	GGGGTCTCACTCACTGCGACTGAAAAACAAGACTACAAAGACCATGACGGTGATTATAA
vca0812_plasmid_flag_rev	Rev	TTATAATCACCGTCATGGTCTTTGTAGTCTTGTTTTTCAGTCGCAGTGAGTGAGACCCC
1807_up_for	Fwd	CTACTCTACCGTCATCGCAGGATTAGCAACGAT
ara_pomA_up_for	Fwd	TGTTTCTCCGGTACCTGCAGGAGGTGTGAAGTGGATTTAGCAACACTGGTTGGTCTTAT
ara_pomA_up_rev	Rev	ATAAGACCAACCAGTGTTGCTAAATCCACTTCACACCTCCTGCAGGTACCGGAGAAACA
pomA_AR_dwn_for	Fwd	AACGTATTCTTGATGTTGATAAAGAGTAGCGGAGCCGACTATTCCGGGGATCCGTCGAC
pomA_AR_up_rev	Rev	GTCGACGGATCCCCGGAATAGTCGGCTCCGCTACTCTTTATCAACATCAAGAATACGTT
1807_dwn_rev	Rev	TACCAGCCTGATGCCGGTACGGCTGAT
ara_flgC_up_for	Fwd	TGTTTCTCCGGTACCTGCAGGAGGTGTGAAATGAGCTTATTCAGCGTATTTAATGTCAC
ara_flgC_up_rev	Rev	GTGACATTAAATACGCTGAATAAGCTCATTTCACACCTCCTGCAGGTACCGGAGAAACA
flgC_AR_dwn_for	Fwd	GTACGCTGCAGATGGGTCAATAAGGATAAGGAGTTAACGTATTCCGGGGATCCGTCGAC
flgC_AR_up_rev	Rev	GTCGACGGATCCCCGGAATACGTTAACTCCTTATCCTTATTGACCCATCTGCAGCGTAC
ara_flaA_up_for	Fwd	TGTTTCTCCGGTACCTGCAGGAGGTGTGAAATGACCATTAACGTAAATACCAACGTGTC
ara_flaA_up_rev	Rev	GACACGTTGGTATTTACGTTAATGGTCATTTCACACCTCCTGCAGGTACCGGAGAAACA
flaA_AR_dwn_for	Fwd	CTTCATTAATGAGCTCAGACGTGGGTATGTAATGAAGGGAATTCCGGGGATCCGTCGAC
